# The response of Arabidopsis to the apocarotenoid β-cyclocitric acid reveals a role for SIAMESE-RELATED 5 in root development and drought tolerance

**DOI:** 10.1093/pnasnexus/pgad353

**Published:** 2023-10-26

**Authors:** Jeanne Braat, Meryl Jaonina, Pascale David, Maïté Leschevin, Bertrand Légeret, Stefano D’Alessandro, Frédéric Beisson, Michel Havaux

**Affiliations:** Aix Marseille University, CEA, CNRS UMR 7265, Bioscience and Biotechnology Institute of Aix Marseille, CEA/Cadarache, Saint-Paul-lez-Durance 13115, France; Aix Marseille University, CEA, CNRS UMR 7265, Bioscience and Biotechnology Institute of Aix Marseille, CEA/Cadarache, Saint-Paul-lez-Durance 13115, France; Aix Marseille University, CEA, CNRS UMR 7265, Bioscience and Biotechnology Institute of Aix Marseille, CEA/Cadarache, Saint-Paul-lez-Durance 13115, France; Aix Marseille University, CEA, CNRS UMR 7265, Bioscience and Biotechnology Institute of Aix Marseille, CEA/Cadarache, Saint-Paul-lez-Durance 13115, France; Aix Marseille University, CEA, CNRS UMR 7265, Bioscience and Biotechnology Institute of Aix Marseille, CEA/Cadarache, Saint-Paul-lez-Durance 13115, France; Universita di Torino, Scienze Della Vita e Biologia dei Sistemi, Torino 10123, Italy; Aix Marseille University, CEA, CNRS UMR 7265, Bioscience and Biotechnology Institute of Aix Marseille, CEA/Cadarache, Saint-Paul-lez-Durance 13115, France; Aix Marseille University, CEA, CNRS UMR 7265, Bioscience and Biotechnology Institute of Aix Marseille, CEA/Cadarache, Saint-Paul-lez-Durance 13115, France

**Keywords:** apocarotenoid, cell division, drought stress, root development, SIAMESE RELATED (SMR)

## Abstract

New regulatory functions in plant development and environmental stress responses have recently emerged for a number of apocarotenoids produced by enzymatic or nonenzymatic oxidation of carotenoids. β-Cyclocitric acid (β-CCA) is one such compound derived from β-carotene, which triggers defense mechanisms leading to a marked enhancement of plant tolerance to drought stress. We show here that this response is associated with an inhibition of root growth affecting both root cell elongation and division. Remarkably, β-CCA selectively induced cell cycle inhibitors of the SIAMESE-RELATED (SMR) family, especially SMR5, in root tip cells. Overexpression of the *SMR5* gene in Arabidopsis induced molecular and physiological changes that mimicked in large part the effects of β-CCA. In particular, the *SMR5* overexpressors exhibited an inhibition of root development and a marked increase in drought tolerance which is not related to stomatal closure. *SMR5* up-regulation induced changes in gene expression that strongly overlapped with the β-CCA–induced transcriptomic changes. Both β-CCA and SMR5 led to a down-regulation of many cell cycle activators (cyclins, cyclin-dependent kinases) and a concomitant up-regulation of genes related to water deprivation, cellular detoxification, and biosynthesis of lipid biopolymers such as suberin and lignin. This was correlated with an accumulation of suberin lipid polyesters in the roots and a decrease in nonstomatal leaf transpiration. Taken together, our results identify the β-CCA–inducible and drought-inducible *SMR5* gene as a key component of a stress-signaling pathway that reorients root metabolism from growth to multiple defense mechanisms leading to drought tolerance.

**Significance Statement**

Carotenoids are natural compounds that fulfill several functions in plants including protection against oxidative stress. Their oxidative cleavage results in a variety of products with important biological functions. β-Cyclocitric acid (β-CCA) is one such compound that triggers stress responses leading to drought tolerance. The molecular mechanisms behind this protection are still elusive. β-CCA–induced drought tolerance was associated with a noticeable inhibition of root growth. A detailed analysis of this phenomenon revealed the selective induction of SMR5, a cell cycle inhibitor. Up-regulation of this gene mimics mostly the effects of β-CCA on root growth, drought tolerance, and gene expression changes. This study identifies SMR5 as a component of a signaling pathway controlling drought tolerance which could constitute a target for engineering stress-resilient plants.

## Introduction

Carotenoids are a large group of compounds constituted by eight isoprene units, the vast majority of which are derived from the linear tetraterpene phytoene (1–4). Modifications of this linear backbone by desaturases, cyclases, hydroxylases, ketolases, and other enzymes give rise to a wide diversity of compounds. The presence of conjugated double bonds in the carotenoid skeleton confers pigment properties to this family of molecules, allowing them to play a variety of physiological functions. Plant carotenoids are mainly known as accessory light-harvesting pigments in photosynthesis, antioxidants, lipid membrane stabilizers, and attractants for pollinators and seed dispersers. Carotenoids are also at the origin of a number of bioactive molecules, including phytohormones such as strigolactones and abscisic acid, by enzymatic or nonenzymatic oxidative cleavage (5, 6). New regulatory functions have recently emerged for some of those cleavage products (apocarotenoids), which relate to plant development and response to environmental stresses (7–9). The volatile β-cyclocitral (β-CC), generated by the oxidation of β-carotene, is one such bioactive compound, which is partly converted in planta into the water-soluble β-cyclocitric acid [β-CCA; 10, 11). β-CC and β-CCA function in plants as molecular signals causing metabolic changes and triggering changes in nuclear gene expression, which lead to an increased tolerance to several biotic and abiotic stresses (12–14). In particular, increasing the internal concentration of β-CCA in plants by exogenous applications was shown to bring about a marked enhancement of drought tolerance (13).

Transcriptomic analyses of plant leaves exposed to volatile β-CC have shown that this apocarotenoid induces cellular defense and detoxification mechanisms (12, 15). More precisely, β-CC triggers the so-called xenobiotic detoxification pathway controlled by TGACG-binding (TGAII) transcription factors interacting with the SCARECROW-LIKE 14 (SCL14) transcription regulator (16). The TGAII/SCL14 complex governs the expression of a variety of detoxification enzymes, which target toxic reactive carbonyls such as those produced by lipid peroxidation (17, 18). Enhancement of the detoxification capacities by β-CC and β-CCA is likely to participate in the enhancement of plant tolerance to stressful conditions, such as drought stress, that produce reactive oxygen species and lead to lipid peroxidation (13, 19).

β-CC was also reported to promote root growth in Arabidopsis, rice, and tomato (20). This effect was maintained under salt stress, suggesting that the regulation of root stem cell behavior by β-CC could increase plant vigor under stressful conditions. This hypothesis prompted us to examine if β-CCA–induced drought stress tolerance is associated with changes in root growth. The results shown in this study indicate that, contrary to what was previously reported for β-CC, β-CCA affects root development by causing a marked inhibition of primary and secondary root growth. The detailed analysis of this phenomenon allowed us to identify a cell cycle–related gene, inducible by β-CCA and water stress, that modulates root development and drought tolerance. The present study describes this new component of the drought stress response of plants.

## Results

### The induction of drought tolerance by β-CCA is associated with root growth inhibition

**Fig. 1.**
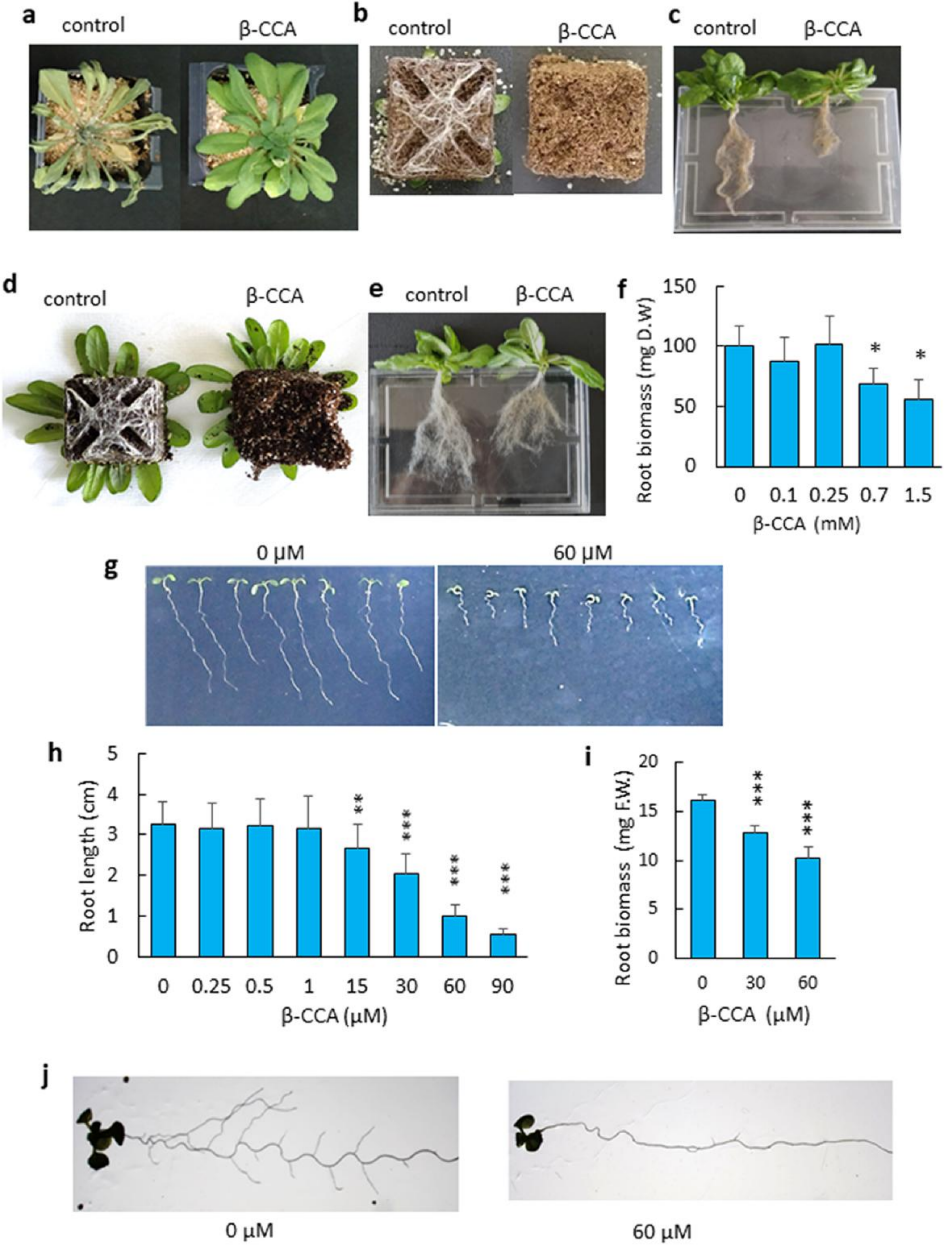
β-CCA inhibits root growth of Arabidopsis plants. a) Picture of Arabidopsis plants exposed to water stress induced by withdrawing watering for 10 days. β-CCA–pretreated plants were watered with 25 mL of a 1.5 mM β-CCA solution prior to water stress. Control plants received pure water instead of β-CCA. b) Picture of the roots at the bottom of the pot after 10 days of water stress, showing the decreased root density with β-CCA treatment. c) Picture of the root system. d–e) Picture of the roots of plants pretreated with 25 mL of 1.5 mM β-CCA or with 25 mL of water (control) and then let to grow with normal irrigation (no water stress). f) Root D.W of plants treated with 25 mL of different solutions of β-CCA (0, 0.1, 0.25, 0.7, and 1.5 mM β-CCA) in the absence of water stress. Data are mean values of three to four measurements + SD. *Different from 0 mM with *P* < 0.05 (Student's t test). g) Picture of the seedlings grown in vitro on Agar with 0 or 60 µM β-CCA. h and i) Root length and biomass as a function of the β-CCA concentration in the in vitro growth medium. Data are mean values of 15 measurements + SD. ** and *** different from 0 µM with *P* < 0.01 and 0.001, respectively (Student's t test). j) Both the primary root and lateral roots of in vitro-grown seedlings were inhibited by β-CCA. D.W, dry weight; F.W., fresh weight.

Arabidopsis plants grown on soil were watered with 1.5 mM β-CCA (or with water for the controls) before stopping watering for 10 days. As expected (13), plants pretreated with β-CCA were much more tolerant to water deprivation than control plants (Fig. 1a). β-CCA–treated plants remained fully turgid, while control plants showed clear signs of leaf dehydration after 10 days of water deprivation. Rather surprisingly, the protective effect of β-CCA was associated with a marked reduction of root length (Fig 1b, c). Root growth inhibition was also observed when well-watered plants were treated with β-CCA and let to grow for 10 days in the absence of any water stress (Fig. 1d, e). Thus, the reduction of root development appears to be a direct effect of β-CCA and does not necessarily require an interaction with drought stress. We previously showed that 1.5 mM citric acid (pH ∼5, same pH as 1.5 mM β-CCA) does not induce drought tolerance, excluding a pH effect (13). Reduction of root biomass was observed when plants were watered with β-CCA solutions at concentrations above 250 µM (Fig. 1f).

The effect of β-CCA on Arabidopsis root growth was further studied in seedlings grown on solid growth medium in Petri dishes. The results shown in Fig. 1g confirm the inhibitory action of the apocarotenoid on root growth, which was observed at concentrations higher than 1 µM (Fig. 1h, i). No stimulatory effect was found in the nM range, contrary to what was previously reported for β-CC (20). The formation of lateral roots was also inhibited by β-CCA (Fig. 1j).

The β-CCA concentration was measured in Arabidopsis by GC-MS. In roots of control plants grown on soil, the β-CCA concentration was low (<15 ng g^−1^ fresh weight), close to the detection limit of our GC system. This concentration is of the order of magnitude of the β-CC concentration in Arabidopsis roots (20). However, this is approximately 10 times lower than the β-CCA levels in leaves (139.53 ± 16.05 ng g^−1^ fresh weight). In plants watered with 1.5 mM β-CCA, the β-CCA concentration in the roots drastically increased to 29.97 ± 11.16 µg g^−1^ fresh weight. We cannot exclude that the low β-CCA concentrations measured in roots of soil-grown control Arabidopsis plants were due to β-CCA losses (or metabolization) during the rather long process of root washing. Accordingly, when the GC analyses were performed on young Arabidopsis seedlings grown in vitro on Agar, the measured concentrations in roots were noticeably higher, 864 ± 263 ng g^−1^ fresh weight.

### Effects of β-CCA on root cell elongation and division

**Fig. 2.**
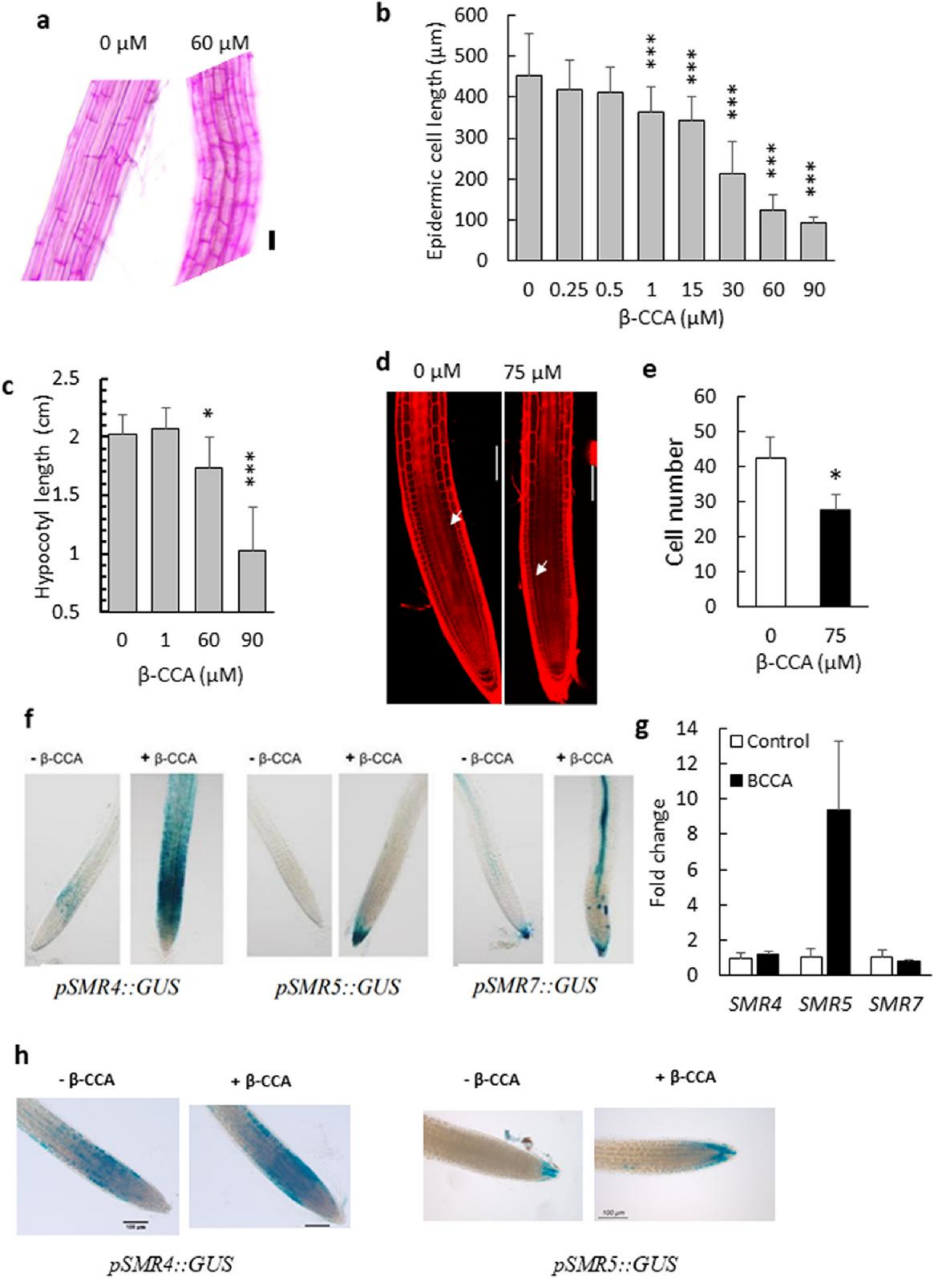
Both cell elongation and cell division in primary roots are perturbed by β-CCA. a) Picture of root epidermal cells in the elongation zone colored with ruthenium red. Seedlings were grown for 6 days on solid Agar medium with 0 or 60 µM β-CCA. b) Quantification of root cell length as a function of the β-CCA concentration. Data are mean values of 20 measurements + SD. c) Effect of β-CCA on Arabidopsis hypocotyl elongation in the dark. d) Picture of the meristem zone in Arabidopsis seedlings visualized by the fluorescence of propidium iodide. Arrows indicate the boundary between cell division and elongation zones. e) Meristem cortex cell number in the division zone of roots of Arabidopsis seedlings treated with 0 or 75 µM β-CCA. Cells were counted from the quiescent center to the boundary of the cell division and elongation zones. Values are means of six measurements + SD. Scale bar in (a) and (d) =100 µm. f) GUS coloration of root tips of the transcriptional GUS reporter lines *pSMR4::GUS, pSMR5::GUS,* and *pSMR7::GUS*. Seedlings were exposed to 0 or 75 µM β-CCA in the growth medium for 6 days. g) qRT-PCR analysis of the relative transcript levels of *SMR4*, *SMR5*, and *SMR7*. Data are normalized to the housekeeping gene *UPL7* and are expressed as average fold changes + SD (3 replicates) compared with the control levels set to 1. h) GUS coloration of root tips of *pSMR4::GUS* and *pSMR5::GUS* seedlings measured 3 h after transfer to a growth medium containing 0 or 75 µM β-CCA. * and *** different from 0 µM with *P* < 0.05 and *P* < 0.001, respectively (Student's t test).

Root length is determined by cell proliferation in the meristem and cell expansion during differentiation. We measured both phenomena by microscopic analyses. First, we measured the effect of β-CCA on cell elongation in Arabidopsis roots colored with ruthenium red. Cell elongation was substantially reduced by β-CCA (Fig. 2a), and the dependence of this inhibition on the β-CCA concentration (Fig. 2b) was quite similar to the plot of root length versus β-CCA concentration as shown in Fig. 1h. We also measured the hypocotyl length of Arabidopsis seedlings grown in the dark, a phenomenon specifically due to cell elongation (21). The results (Fig. 2c) show that β-CCA leads to shorter hypocotyls, confirming that the apocarotenoid is an inhibitor of cell expansion.

We also used confocal microscopy and fluorescence staining of cell walls with propidium iodide to investigate the effects of β-CCA on cell division in the root meristem (Fig. 2d). β-CCA markedly decreased the size of the root apical meristem. The average number of meristematic cortex cells in the apical meristem of β-CCA–treated seedlings was around 65% of control seedlings (Fig. 2e). This indicates a reduction in the cell division rate as a response to β-CCA.

Cell proliferation occurs as a result of periodic activation of cyclin-dependent kinases (CDKs) by different cyclins (CYCs), ensuring the transition from one phase of the mitotic cycle to another (22–24). To characterize further the impact of β-CCA on root cell division, we checked the effect of β-CCA on the promoter activity of a panel of mitotic marker genes fused to a gene encoding the GUS reporter (25). These lines are markers of the G1 and S phases of the interphase (CYCA3 and CYCD) and of the second gap phase G2 and the M mitotic phase [CYCA2 and CYCB; 26–28). The color patterns of those GUS lines for cyclins are shown in Fig. S1. No significant change was found for most cyclins (CYCA3;1, CYCD3;3, CYCB1;2, CYCA2;3, CYCA3;2, CCSS2A1) in response to β-CCA. An increase, rather than a decrease, in GUS coloration with the β-CCA treatment was observed for CYCD6;1 and CYCB1;1. The effect of β-CCA on the expression of other cyclins and of CDKs will be shown below with the transcriptomic data.

CYC–CDK activity is regulated by several mechanisms, including transcriptional regulation, proteolysis, and interactions with CDK inhibitors (29). CDK inhibitors are crucial for plant development, particularly during the transition from the mitotic cell cycle to endoreplication (30, 31). We examined the expression of the plant-specific CDK inhibitors SIAMESE (SIM) and SIAMESE-RELATED (SMR), which can interact with and inhibit all CDKs (30, 32–34), using promoter-driven GUS reporter lines (25). The expression of SIM did not respond to β-CCA (Fig. S1). In contrast, SMR4, SMR5, and SMR7 GUS reporter lines showed the most remarkable responses (Fig. 2f). Although the SMR protein family is rather large (29), SMR5 and to a lesser extent SMR4 and SMR7 have been previously shown to be the most responsive SMRs to environmental factors (35, 36).

We also analyzed the expression of the three *SMR* genes by qRT-PCR. The *SMR5* transcripts (AT1G07500) accumulated when Arabidopsis was treated with β-CCA, whereas no significant change was observed for the *SMR4* (AT5G02220) and *SMR7* (AT3G27630) transcripts (Fig. 2g). This differential response of the three *SMR*s was also found in the RNA sequencing (RNA-seq) analysis (see below). It is possible that the combination of low expression and very localized expression pattern of *SMR4* and *SMR7* makes them difficult to be monitored in the root samples harvested for the qRT-PCR analyses. The stability of the mRNA of the three *SMR*s may also be different.

The induction of *SMR4* and *SMR5* is an early response to β-CCA that occurred before root growth was markedly inhibited. Indeed, when seedlings grown for 7 days in the absence of β-CCA were transferred to a medium containing 75 µM β-CCA, the expression of both *SMR* genes was enhanced after a short exposure of 3 h to β-CCA (Fig. 2h).

The gene expression data of Fig. 2 show that β-CCA induces the *SMR5* gene. Considering the known function of SMRs in cell division, this is potentially an important component of the root growth inhibition by β-CCA and possibly also of the enhancement of plant drought tolerance.

### Role of SMR4 and SMR5 in root growth

To examine the possible role of SMR expression in root growth and drought tolerance, we investigated the responses of SMR-deficient and SMR-overexpressing Arabidopsis lines to water stress and β-CCA.

Suppression of *SMR4*, *SMR5*, and *SMR7* in a triple mutant (*smr4 smr5 smr7*), previously described in (37), had virtually no effect on shoot and root growth (Fig. S2). Neither the shoot morphology and size (Fig. S2a, b) nor the root length and biomass (Fig. S2c–f) were affected by the lack of the three SMRs. This was observed under different growth conditions: on soil, on sand, and in vitro on solid Agar medium. Also, the response to β-CCA was not impaired in the *smr4 smr5 smr7* triple mutant: β-CCA inhibited root growth (Fig. S2f) and enhanced drought tolerance (Fig. S2g). Triple mutant plants were also exposed to water deprivation without the β-CCA treatment (Fig. S3). Their drought tolerance did not significantly differ from that of wild type (WT): leaf RWC decreased to around 60% in both genotypes after 8 days of water deprivation.

**Fig. 3.**
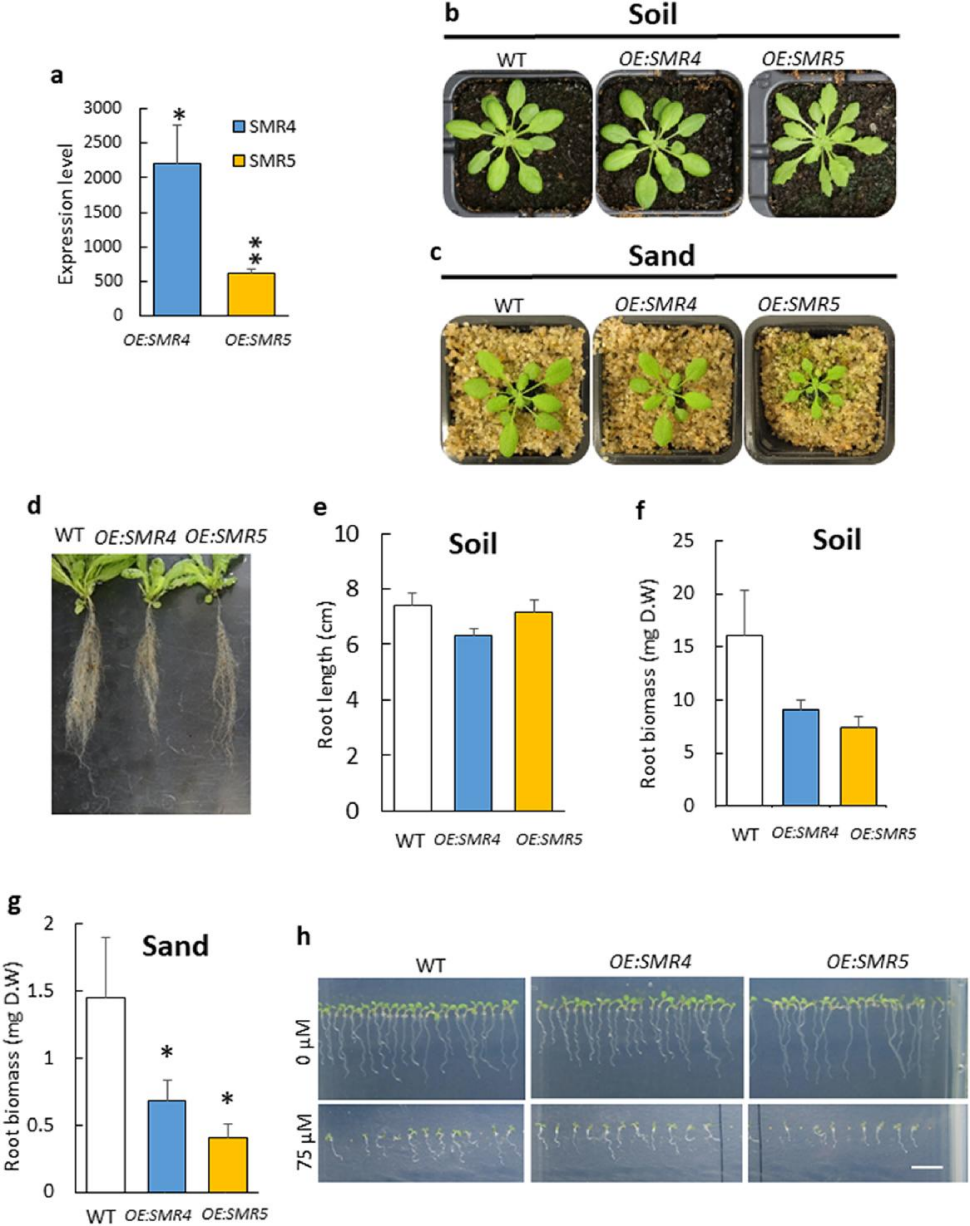
Growth of plants overexpressing *SMR5* or *SMR4*. a) Expression levels of *SMR4* and *SMR5* in leaves of the corresponding overexpressing lines compared with WT levels set to 1. b and c) Picture of plants (WT, *OE:SMR5* and *OE:SMR4*) aged 5 weeks or 4 weeks grown on soil or on sand, respectively. d) Root system of plants grown on soil for 6 weeks. e) Length of the root system of plants grown on soil for 6 weeks (3 replicates + SD). f and g) Root dry weight of plants grown on soil or on sand (3 to 7 replicates + SD). h) In vitro growth of the plants on Agar with or without 75 µM β-CCA. Scale bar, 1 cm. * and ** different from WT at *P* < 0.05 and 0.01, respectively (Student's t test).

The SIM/SMR family is a large family, composed of at least 17 members in Arabidopsis (29), and therefore, we cannot exclude a compensation mechanism in the triple SMR mutant involving functional substitution by several members of the SIM/SMR family. There are also seven genes encoding KRP proteins, which constitute another class of CDK inhibitors (29). To overcome the high redundancies in this regulation, we adopted an overexpression approach. Two lines overexpressing either *SMR4* or *SMR5* (designated as *OE:SMR4* or *OE:SMR5*), previously described (35), were available for this study. Both lines exhibited high levels of *SMR* expression compared with the WT level (Fig. 3a).

When plants were grown on soil in our growth conditions, shoot development of the *SMR* overexpressors did not strongly differ from that of WT (Fig. 3b). There was no reduction in the fresh weight of shoot biomass of the *OE:SMR*s compared with WT (Fig. S4). We observed a change in the shoot morphology of the *OE:SMR5* overexpressor which exhibited serrated leaves, as also reported previously in (35). This seems to be a characteristic of plants exhibiting high SMR levels, as previously observed in a strong *SMR1* overexpressor (36).

Root development was significantly affected in *OE:SMR* plants, with a decrease in root biomass (dry weight), particularly in *OE:SMR5* relative to WT (Fig. 3d, f). Root growth was slightly less inhibited in *OE:SMR4* compared with *OE:SMR5* (Fig. 3f). Contrary to root biomass, the total length of the root system was not reduced in the *OE:SMR* lines (Fig. 3d, e), indicating only partially overlapping changes with those induced by β-CCA (Fig. 1). When plants were grown on sand, the growth phenotype of *OE:SMR4* and *OE:SMR5* was more marked, with root and shoot sizes being reduced in both overexpressors compared with WT (Fig. 3c, g). Thus, the impact of *SMR4* and *SMR5* overexpression appeared to be dependent on the growth conditions. This is confirmed when seedlings were grown in vitro on solid medium (Fig. 3h): primary root growth was slightly inhibited in *OE:SMR4*, not in *OE:SMR5*.

### Drought tolerance associated with *SMR4* or *SMR5* overexpression

**Fig. 4.**
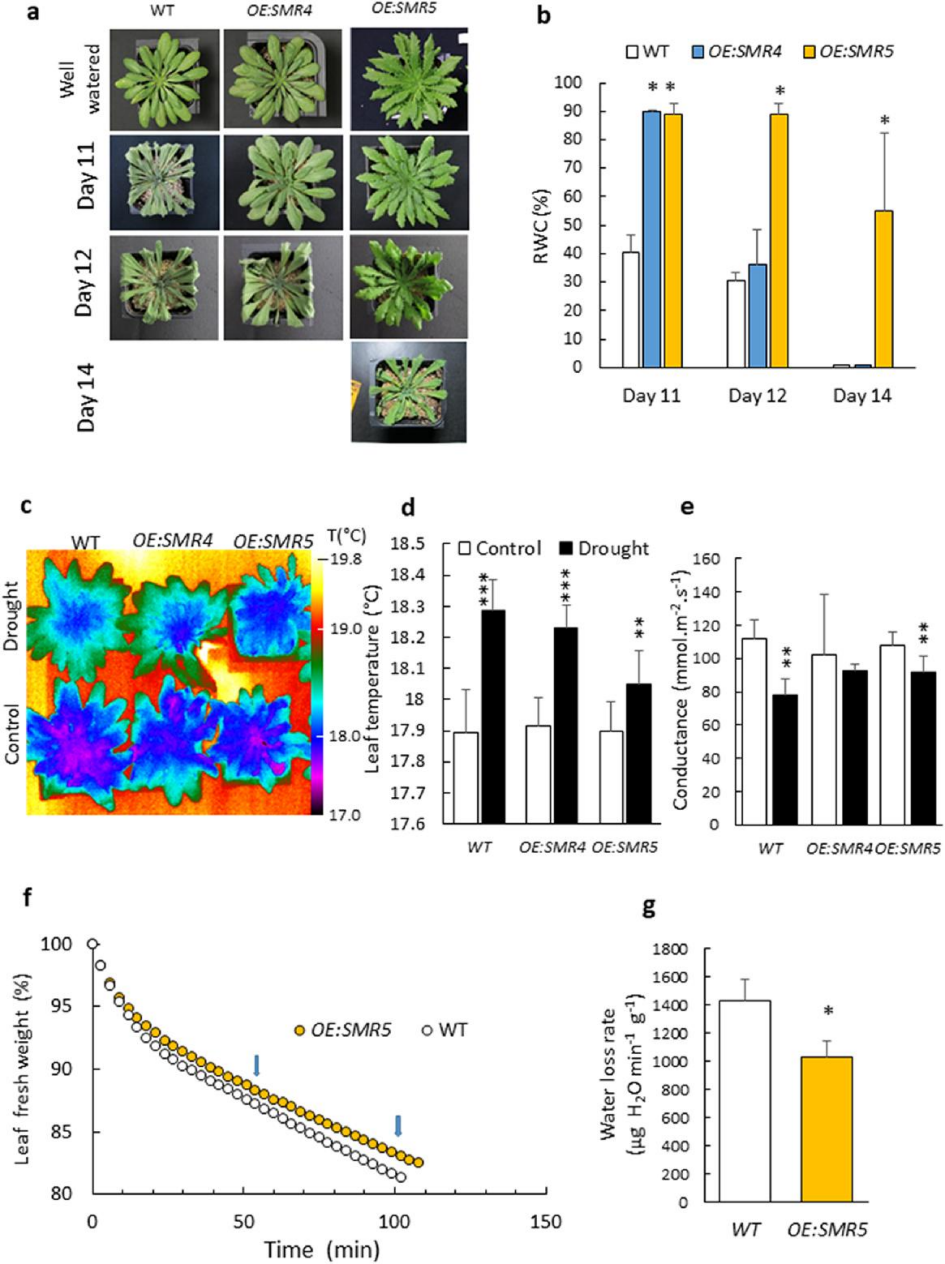
SMR4-overexpressing or SMR5-overexpressing plants are tolerant to drought stress. a) Picture of the plants (WT, *OE:SMR5* and *OE:SMR4*) water-stressed for 10, 12, and 14 days. Water stress was imposed by stopping watering. Absence of picture means that the plants died. CTRL corresponds to well-watered plants. b) Leaf RWC (mean values of three separate experiments + SD). c) IR images of leaf temperatures in control plants and plants exposed to drought stress for 6 days. d) Average temperature of mature leaves. Data are mean values of four different plants + SD. e) Porometric measurements of stomatal conductance (6 replicates + SD). f–g) Water losses by excised plants in the dark. f) Shows typical plot of the time course of leaf weight changes in the dark. Data of (g) (mean values of 4 measurements + SD) were calculated from the linear part of the curve indicated by the arrows. *, ** and *** different from WT or control at *P* < 0.05, 0.01 and 0.001, respectively (Student's t test).

WT and the two *SMR* overexpressors were exposed to drought stress imposed by withholding watering. Strikingly, *OE:SMR5* and *OE:SMR4* were much more tolerant to water stress than WT (Fig. 4a). After 10 days of water deprivation, WT plants were dehydrated, with the relative water content (RWC) dropping to ca. 40%, while both overexpressors remained turgescent, with an RWC above 90% (Fig. 4b). Drought tolerance of the *OE:SMR4* line was intermediate between WT and *OE:SMR5*: RWC decreased to the WT level at day 12. Strikingly, a prolonged water stress of 14 days was necessary to observe stress consequences in *OE:SMR5* plants, with RWC decreasing to around 50%.

To confirm the involvement of SMR4 and SMR5 in drought tolerance, we generated additional *SMR4*-overexpressing and *SMR5*-overexpressing transgenic lines. Figure S5 presents a selection of independent transgenic lines with different levels of *SMR4* or *SMR5* overexpression (Fig. S5b) after 12 days of water deprivation (Fig. S5a). All the lines were more tolerant to water stress than WT, remaining turgescent and keeping high RWC values under very harsh conditions (Fig. S5e). Root biomass was reduced in all *OE:SMR* lines compared with WT (Fig. S5c, d). It is to be noted that the majority of transgenic plants had serrated leaves.

Application of β-CCA on *SMR*-overexpressing lines did not increase further their resistance to water stress (Fig. S6). After 15 days of water deprivation, RWC dropped on average to around 50% in *OE:SMR5* and around 30% in *OE:SMR4* independently of β-CCA (Fig. S6c). Thus, the β-CCA effect and the *SMR4*-overexpression or *SMR5*-overexpression are not cumulative, suggesting that β-CCA and SMR5 could be in the same pathway.

### *SMR* overexpression does not decrease stomatal conductance

Stomatal closure is a typical response of plants exposed to water stress conditions in order to limit water losses by transpiration (38, 39). We analyzed the stomatal functioning in WT and *OE:SMR* plants using two complementary techniques, infrared (IR) imaging and porometry.

Leaf temperature varies with the transpiration rate: opening of the stomata results in evaporative cooling and a decrease in leaf temperature, which can be monitored by thermal imaging (40, 41). Figure 4c shows the images of plant temperature obtained with an IR camera. No difference was observed in leaf temperature between plants of the three genotypes under control conditions. Then, we exposed plants to a mild water stress induced by stopping watering for 4 days to avoid complete stomatal closure (like in Fig. 1a or Fig. 4a). Water stress induced a rise in leaf temperature reflecting a decrease in stomatal aperture in WT plants (Fig. 4c, d). The effect of drought was more marked in WT and *OE:SMR4* compared with *OE:SMR5*, likely due to the high drought tolerance of the latter genotype. Therefore, the IR images support that stomatal transpiration is not reduced in the *OE:SMR* plants compared with WT plants, both under standard and stress conditions.

Porometry measurements were also conducted to evaluate the stomatal conductance by measuring the rate of water efflux from the leaf abaxial side. Under control conditions, stomatal conductance was similar in WT and *OE:SMR* plants (Fig. 4e), in agreement with Fig. 4c, d. Mild water stress conditions decreased stomatal conductance in WT and *OE:SMR* leaves, consistently with the increased temperature of leaves under those conditions. We can conclude that the increase in drought tolerance of *OE:SMR* plants does not rely on a reduction of stomatal aperture relative to WT.

Cuticular transpiration was measured by monitoring water losses from excised rosettes placed in the dark. Upon transfer of the plant rosettes from light to darkness, there was a rapid loss of water for ca. 30 min followed by a linear decrease in the plant mass (Fig. 4f). The first phase reflects the loss of water through the stomata, which are closing in the dark (42). The second phase reflects the cuticular losses of water after closure of the stomata. The second, linear phase was slowed down in the *OE:SMR5* plants (−28%, Fig. 4f, g) and, to a lesser extent, in the β-CCA–exposed plants compared with control WT rosette (−16%, Fig. S7). This indicates a decrease in nonstomatal leaf transpiration by both β-CCA treatment and *SMR5* overexpression.

### Water stress induces *SMR* expression and inhibits root growth

**Fig. 5.**
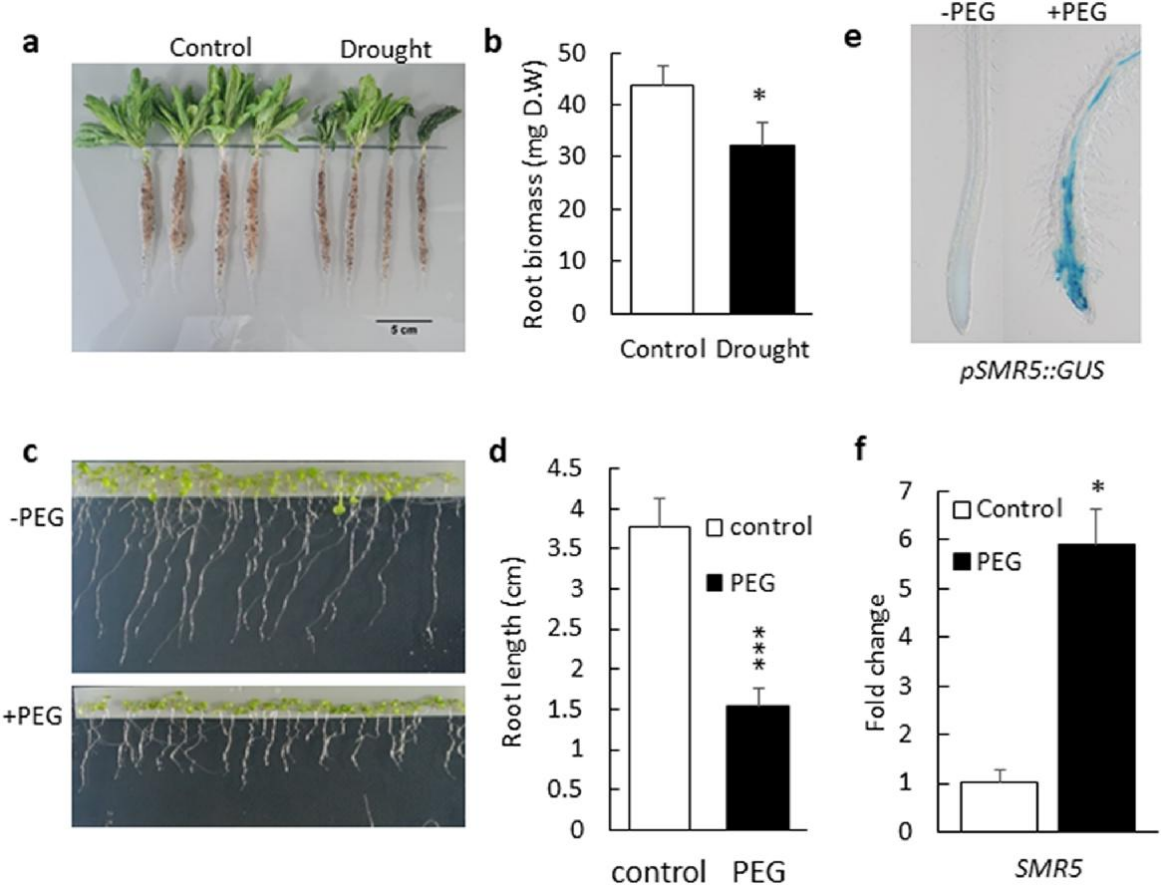
Effects of drought and osmotic stress on the Arabidopsis root system. a) Root system of control WT plants or drought-stressed plants grown on soil. b) Root dry weight. Values are means of four measurements + SD. c and d) Root length of plants grown in vitro on Agar in the presence or absence of PEG 8000. Seedlings were grown on control medium for 3 days on nylon stripes before transfer to PEG-enriched medium for 4 days. e) Expression pattern of *SMR5* visualized by GUS coloration in the root tips of *pSMR5::GUS*. The plants were grown on Agar in the presence or absence of PEG 8000. f) qRT-PCR analysis of the transcript levels of *SMR5*. Data are mean values of three replicates + SD. They were normalized to the house keeping gene *UPL7* and to the control levels set to 1. * and *** different from WT at *P* < 0.05 and *P* < 0.001, respectively (Student's t test).

We previously showed that drought stress conditions induce β-CCA accumulation in Arabidopsis plants (13). Drought is also known to change root architecture [e.g. 43, 44), and this phenomenon is confirmed here by the reduction of root length in Arabidopsis plants exposed to drought compared with well-watered conditions (Fig. 5a). Root dry weight was reduced by 25% in drought-stressed Arabidopsis plants (Fig. 5b). Similarly to plants grown on soil, Arabidopsis seedlings exposed to water deficit induced by adding PEG-8000 to the solid growth medium had shorter roots compared with control seedlings (Fig. 5c, d). This was associated with a substantial induction of *SMR5* (Fig. 5e, f). Thus, water stress is an environmental condition where the correlation between *SMR* induction and root growth inhibition found in β-CCA–treated plants is also observed.

### Large overlaps between the transcriptomic responses to β-CCA treatment and to *SMR5* overexpression

**Fig. 6.**
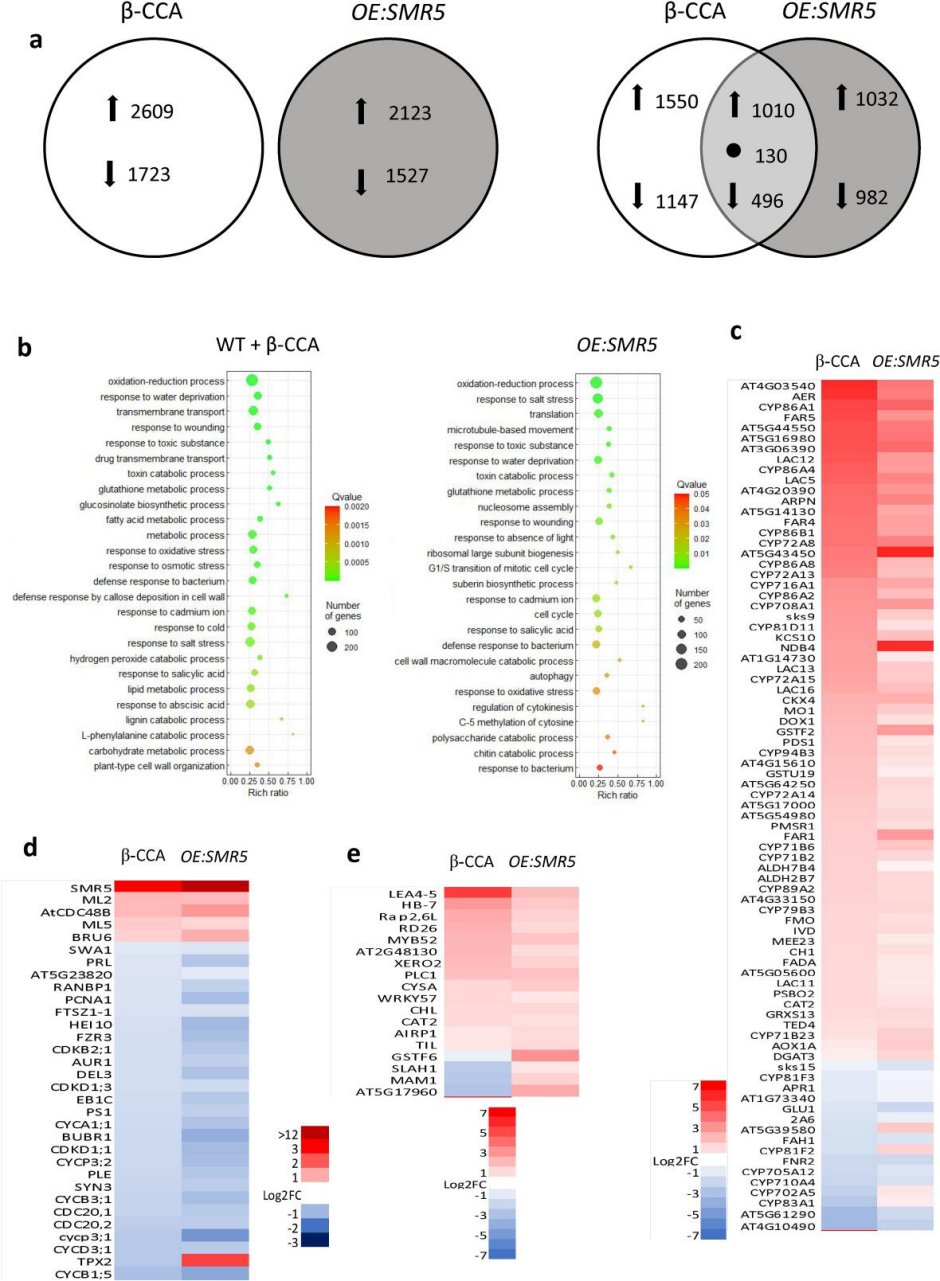
Transcriptome of Arabidopsis plants treated with β-CCA or overexpressing *SMR5* compared with untreated WT plants. a) Left: Number of DEGs (DESeq, *P*-adjusted <0.05, |log_2_ (fold change)| ≥0) in roots of β-CCA treated seedlings versus untreated WT seedlings and of *OE:SMR5* seedlings versus WT seedlings. Arrows indicate up-regulation and down-regulation. Data are mean values of three separate experiments. Right: Venn diagram of up-regulated and down-regulated genes. Arrows indicate up-regulation and down-regulation, and • indicates genes that changed in the opposite manner in the β-CCA–treated seedlings and in the *OE:SMR5* seedlings (49 down-regulations and 81 up-regulations in *OE:SMR5* and vice versa in β-CCA). b) Gene ontology biological process (GOPB) enrichment bubble plots. Functional categories of the most affected genes by β-CCA or *SMR5* overexpression are shown. Bubble color and size indicate the Q value and gene number enriched in the biological process. c–e) Heat maps of differentially expressed genes in roots of β-CCA–treated plants or plants overexpressing *SMR5* compared with untreated control WT plants. Genes are related to c) oxidoreduction processes, d) cell division, and e) water deprivation. *P* < 0.05. Log_2_ (fold change) ≥0.

We performed a transcriptomic analysis of root tips from the control and β-CCA–treated WT plants by RNA-seq. Around 4300 genes were differentially expressed between the two types of plants (Fig. 6a), with about 2600 genes being induced by β-CCA and more than 1700 genes being down-regulated compared with untreated control plants. Classification of the differentially expressed genes in functional categories (Fig. 6b) revealed interesting features of the β-CCA effects on gene expression. The highest numbers of genes responsive to β-CCA were found in categories that are related to the responses to water deprivation/osmotic stress/oxidative stress, to oxidoreduction processes (including detoxification processes), to transmembrane transport, to cell wall, and to different metabolic processes, reflecting a global response to stress.

RNA-seq analysis was also conducted on root tips of the *SMR5* overexpressor. The number of genes induced or repressed compared with WT was above 2100 and 1500 genes, respectively (Fig. 6a). There was a substantial overlap in the transcriptomic responses to β-CCA and to *SMR5* overexpression with about 1000 up-regulated and 500 down-regulated genes in common (Fig. 6a). Interestingly, there was a noticeable concordance in the functional categories of the most affected genes by *SMR5* overexpression and by β-CCA, with enrichment in genes related to oxidoreduction processes, responses to water deprivation/osmotic stress/oxidative stress and cell wall in both conditions versus WT (Fig. 6b).

The heat maps of genes linked to oxidoreduction processes (Fig. 6c), cell division (Fig. 6d), and water deprivation (Fig. 6e) confirm the strong homology between the gene signature of β-CCA–treated plants and *SMR5*-overexpressing plants. Almost all the genes induced by β-CCA were also induced in *OE:SMR5*, and similarly all the genes down-regulated by β-CCA were down-regulated by *SMR5* overexpression in each gene category. Interestingly, a number of genes belonging to the “cell division” category, including a number of cyclins and CDKs, were down-regulated in the β-CCA–treated plants and *OE:SMR5*, consistently with the inhibitory effect on root cell division (Fig. 6d). *SMR5* was one of the most induced genes by β-CCA in this category. In addition to the strong induction of *SMR5* by β-CCA (Fig. 6), *SMR8* was induced on the order of two-fold (Fig. S8a), and this was confirmed by qRT-PCR (Fig. S8b). The latter effect could be important because the basal expression level of *SMR8* is much higher than the *SMR5* level (TPM [Transcripts Per Kilobase Million] of 13.8 and 0.4, respectively), and therefore a two-fold increase in *SMR8* transcripts could have a very significative effect on the total *SMR* mRNA pool. Thus, *SMR8* could be a good candidate for the compensation of the lack of *SMR* genes in *smr4 smr5 smr7*. However, qRT-PCR analysis of *SMR8* expression in the triple mutant revealed no difference with WT (Fig. S8c), undermining our hypothesis. We also analyzed the expression of two genes, *GPAT5* and *WRKY24*, strongly induced by β-CCA and *SMR5* overexpression in the transcriptomes (Fig. S8d): their expression levels were significantly reduced in *smr4 smr5 smr7* compared with WT, indicating partial deregulation of gene expression in the triple mutant.

There was a high concordance in the induction/repression of genes related to oxidoreduction processes between β-CCA–treated plants and *OE:SMR5* (Fig. 6c). Finally, both β-CCA and *SMR5* up-regulation induced many genes responsive to water deprivation (Fig. 6e).

### Effect of β-CCA and SMR5 on root suberin and leaf cuticular lipids

**Fig. 7.**
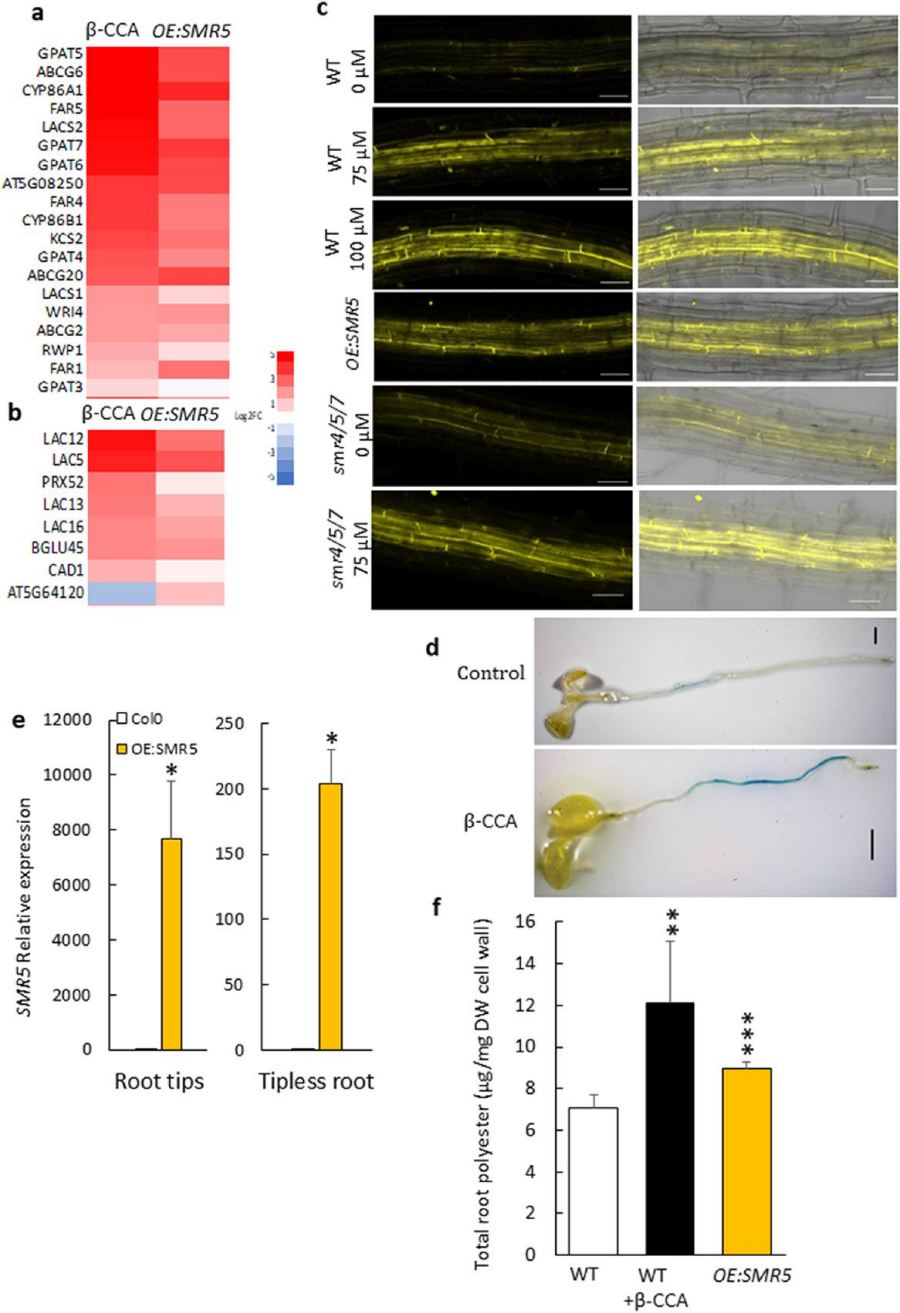
Root suberin in Arabidopsis plants treated with β-CCA or overexpressing *SMR5*. Heat maps of differentially expressed genes related to suberin (a) and lignin (b) biosynthesis. c) Suberin visualization by the fluorescence of fluorol yellow (left side). Scale bars, 50 µm. Right side: merged fluorol yellow fluorescence and transmission light images. Suberin accumulation is also shown for a *smr4 smr5 smr7* triple mutant. d) Root coloration of transcriptional GUS reporter lines *pSMR5::GUS* after 6-day treatment. Scale bars, 1 mm. e) Relative expression level of the *SMR5* gene in different part of the roots (tips and tipless roots) of *OE:SMR5*. f) Total root polyesters. Data are mean values of four to six separate measurements + SD. *, **, and *** different from WT at *P* < 0.05, 0.01, and 0.001, respectively (Student's t test).

The transcriptome of β-CCA–treated plants and *SMR5*-overexpressing plants revealed modifications of the expression of genes involved in the biosynthesis of the cell wall–associated biopolymers, suberin and lignin (Fig. 7a, b) and in the deposition of callose in cell walls (Fig. 6b). Changes in those compounds can have significant effects on root and leaf hydraulics (45). Among the genes known to be involved in suberin/cutin biosynthesis (46), *LACS,* several *GPAT* genes (*GPAT 4, 5, 6, 7*), several *FAR*, *ABCG*, and *CYP* genes were noticeably up-regulated (Fig. 7a).

Suberin was imaged in roots using the fluorescent probe fluorol yellow (47). The intensity of the fluorescence signal was much stronger in the roots of β-CCA–treated seedlings and in *OE:SMR5* seedlings compared with WT (Fig. 7c). It is important to note that *SMR5* induction did not occur in the root tips only, since *SMR5* up-regulation was also found in differentiated root tissues that accumulated suberin (Fig. 7d). This suberization of the root tissues was confirmed by GC-MS analyses: total lipid polyesters of roots increased by a factor of almost 2 from 7.3 to 12.2 µg mg^−1^ cell wall after β-CCA treatment of the plants (Fig. 7f). Most polyesters were increased, but the strongest relative enhancement was observed for the 22:0 and 24:0 fatty acids, the 24:0 ω-OH, 18:1 ω-OH hydroxy fatty acids, and the 24:0, 18:1, and 18:2 α-ω dicarboxylic acids (DCAs) (Fig. S9). The total lipid polyester levels were also increased in *OE:SMR5* roots relative to control WT (about +17%). The most enhanced monomers were the 18:1 and 16:0 ω-hydroxy fatty acids and 18:2 DCA (Fig. S9). Consistently with their drought tolerance (Fig. S2), the roots of *smr4 smr5 smr7* triple mutant lines were able to accumulate suberin in response to β-CCA (Fig. 7c).

It is possible that cuticular lipid polyesters are also up-regulated in leaves by β-CCA. The decrease in leaf cuticular transpiration shown in Fig. 4 could suggest an increase of cuticular lipids. However, this was not observed when cutin and waxes were analyzed by GC-MS: no effect on the cutin levels was found in response to the β-CCA treatment or the *SMR5* overexpression (Fig. S10). Wax coverages did not increase in either of these plants (Fig. S11).

## Discussion

### β-CCA is a root growth inhibitor

This work has shown that the apocarotenoid β-CCA, which accumulates in plants under abiotic stress conditions (11, 13), has negative effects on root growth by inhibiting both cell division and expansion. It is well established that carotenoid-derived phytohormones, such as abscisic acid and strigolactones, shape plant root architecture under specific environmental conditions (48–50). Recently, a number of bioactive metabolites derived from ROS (Reactive Oxygen Species)-mediated or enzymatically catalyzed oxidation of carotenoids, such as β-CC, retinal, (iso-)anchorene, and zaxinone, have been identified as new root growth regulators (51). They have been reported to act as stimulators of root growth (20, 52–54), except isoanchorene that, in contrast, inhibits primary root growth (55). β-CCA is another apocarotenoid that down-regulates root development, affecting both primary and secondary roots. This work thus reinforces the recent view that a network of carotenoid-derived metabolites plays essential roles in root development and in shaping root system architecture (51).

A previous report on the stimulatory effect of volatile β-CC on root growth (20) may seem contradictory to the fact that β-CC is converted into a root inhibitor (β-CCA) in planta (13). We performed the β-CC treatments reported in (20) under our conditions (MS/10, 0.5% sucrose), and we found that volatile β-CC leads to an accumulation of β-CCA in the leaves (as expected) and also in the roots (Fig. S12c). At the β-CC concentration used [25 µM in Agar medium, not in direct contact with the seedling roots, as in (20)], we observed an inhibition, rather than a stimulation, of root growth, consistently with the root accumulation of β-CCA (Fig. S12a, c). Arabidopsis roots were also directly exposed to β-CC by adding 750 nM β-CC to the Agar medium. In this case also, β-CCA accumulated in the roots (Fig. S12e) and root growth was inhibited (Fig. S12d, f). To reconcile these findings with the results of Dickinson et al. (20), we have to assume that the uptake of volatile β-CC by Arabidopsis seedlings and the in vivo transformation of β-CC into β-CCA are dependent on the growth conditions. The key environmental factors involved in this modulation remain to be identified.

From a more general point of view, our results fit with the idea that modulating the content of carotenoids and of some of their derivatives could provide new tools to enhance plant yield and/or resilience (56). Moreover, cell division and expansion at the root tip are known to be reduced when water availability drops (43, 44), as confirmed here by the inhibition of Arabidopsis root growth by water/osmotic stress conditions. Consequently, we can propose that the stress-induced, root-inhibiting β-CCA signal plays a role in the root growth response to drought.

### β-CCA inhibits the root cell division mechanism

This work provides clues on the mode of action of β-CCA on root growth and drought tolerance. The expression of components of the cell division mechanism, including several CDKs and CYCs, was lowered in root tips by β-CCA, whereas CDK-CYC inhibitors, especially SMR5, were up-regulated. This concomitant and opposite change in root cell division activators and inhibitors by β-CCA is expected to negatively affect root growth. Accordingly, accumulation of SMR1 in Arabidopsis was previously reported to cause root growth inhibition (36). In maize, expression analyses of *SMR* genes indicated that they are strongly associated with abiotic stresses including salt stress and osmotic stress (57). On the other hand, cyclin expression is a limiting factor for root growth, and overexpression of *CYC1* was found to accelerate root growth (58). Conversely, the inhibition of *CYCP3;1* expression by the brassinosteroid signaling was reported to inhibit root growth (59), and a mutant deficient in CYCD4;1 exhibited a decreased density of lateral roots (60). Reduction of root growth rate by salt stress was associated with a decrease in the kinase activity of CDKs (61), and loss of CDKC;2 increased drought tolerance in Arabidopsis (62). Those previous observations indicate that the β-CCA–induced perturbations of the cell division machinery can not only inhibit root growth but it can also lead to an enhancement of drought tolerance. Our results and previous observations (35, 36, 63) also indicate that, compared with other *SMR*s, *SMR5* expression is particularly responsive to environmental stresses.

### SMR cell cycle inhibitors promote drought tolerance

*SMR5* overexpression affected the expression of a large number of genes and led to drought tolerance. There was a strong overlap in the transcriptomic responses of Arabidopsis plants treated with β-CCA or overexpressing *SMR5*. Thus, *SMR5* expression partially mimics the effects of β-CCA in both gene expression changes and physiological responses. Interestingly and, similarly to β-CCA (13), *SMR5* up-regulation induced the expression of a number of drought stress marker genes (*RAP2.6, RD26, LEA4-5*, *HB7*, …). Thus, high *SMR5* expression is somehow perceived by the plant as a drought stress signal.

The transcriptomes of β-CCA–exposed roots and *SMR5*-overexpressing roots provide some indications on how plants became resistant to severe drought stress. In leaves, a major transcriptomic effect of β-CC and β-CCA was the induction of the xenobiotic detoxification pathway (13, 16). This pathway detoxifies harmful and reactive compounds generated during stress, such as reactive carbonyl species generated from lipid peroxidation, by a network of detoxifying enzymes, limiting cellular injuries and restricting propagation of oxidative stress (19). Our transcriptomic analyses show that many genes of the xenobiotic response (e.g. *AER*, two *ALDH* genes, several *GSTU* genes, and many *CYP* genes encoding cytochromes P450, …) were induced in roots by β-CCA and SMR5. This effect can contribute to drought tolerance by preserving root cell integrity (64).

Higher drought tolerance of plants with shorter or less dense root system is somewhat counterintuitive. However, deep roots for water acquisition confer advantages for plants growing under conditions where deep-stored water is available (65), obviously not for plants that must capture shallow water or that are grown in containers. Terrestrial plants evolved in environments that favor phenotypes with aggressive root proliferation, but it has been argued that parsimonious root phenotypes with reduced crown root production and less lateral roots are advantageous for drought resistance in high-input agroecosystems (66). van Oosterom et al. (67) showed that maize genotypes capable of maintaining transpiration at low root mass show a better adaptation to drought stress. In Arabidopsis too, there are examples where drought tolerance is associated with reduced root development (68–71). In our experiments, we measured soil moisture at the top and the bottom of the pots at different stages of water stress (0, 7, and 12 days). The changes in soil moisture were very similar for water-stressed WT and *OE:SMR5* plants (Fig. S13). Therefore, we can exclude that the high tolerance of *OE:SMR5* plants relative to WT is attributable to a difference in soil drying during the stress experiment.

We previously showed that neither β-CC nor β-CCA modify stomatal behavior: treatments of plants with those compounds did not trigger stomatal closure (12, 13) and did not change stomatal density (13), and the response of stomata to water stress was unchanged compared with control, untreated plants (13). This is confirmed here by porometric measurements of stomatal conductance and by IR imaging of leaf temperature in the β-CCA–treated plants and *SMR5-*overexpressing plants. We can conclude that the increased tolerance to drought stress by β-CCA or *SMR5* expression does not rely on changes in stomatal transpiration. However, we observed here that both SMR5 and β-CCA lowered the nonstomatal water losses of leaves. Although the rate of the latter mechanism is small compared with stomatal transpiration, the residual transpiration (by closed stomata and cuticle) can play a significant role under drought stress conditions.

The effect of β-CCA and SMR5 on nonstomatal transpiration can be linked to the induction of genes involved in several metabolic pathways leading to cell wall–associated biopolymers such as cutin, suberin, or lignin. The leaf cuticle constitutes a hydrophobic thin layer that can modulate nonstomatal water losses and thus contribute to drought tolerance (72, 73). However, wax and cutin amounts are not always correlated with cuticular transpiration (74). In our study too, we could not correlate cutin and wax levels with the changes in cuticular transpiration. Therefore, other parameters of leaf hydraulics must play a role in the lowering of nonstomatal transpiration by β-CCA and *SMR5* expression, such as the accumulation of other components of the cuticle, like the nonsaponifiable cutan polymer (75) and/or to the establishment of internal barriers regulating the flow of water from the leaf tissues to the atmosphere (76). Interestingly, a number of aquaporin genes (*PIP1;1, PIP1;2, PIP2;2*, and *PIP1;5*) were found to be down-regulated in the leaf transcriptome of Arabidopsis plants exposed to volatile β-CC (12). The exact causes of the decrease in nonstomatal transpiration remain, however, to be determined.

The β-CCA–exposed roots exhibited high levels of suberin monomers compared with control WT plants. Suberin is a lipophilic polyester associated to the cell walls in some tissues such as the endodermis and the periderm of roots and functions as a diffusion barrier for water, gases, and solutes (77). Suberin deposition in roots is enhanced under environmental stress conditions including drought stress, decreasing root hydraulic conductivity (78) and enhancing drought tolerance (79). Consequently, suberin accumulation in Arabidopsis roots by β-CCA and *SMR5* up-regulation could participate in the enhancement of drought tolerance by limiting water losses during water stress. The transcriptomic data also reveal β-CCA–induced and SMR5-induced changes in the expression of genes related to the biosynthesis of other biopolymers, such as callose and lignin, which could further reinforce this protective mechanism against drought stress (80, 81).

## Conclusions

Through the study of the β-CCA effect, we have identified an SMR gene that plays a role in drought tolerance and could therefore constitute a new target for improving plant resilience to climatic stresses. Although SMRs are inhibitors of cell division, the overexpression of *SMR5* or *SMR4* had modest effects on shoot growth. In contrast, the effect on roots was much more pronounced. Similarly, the growth of the aerial parts of Arabidopsis plants was not affected by β-CCA (13), whereas roots were noticeably impacted (this study).

It appears from our results that reorientation of root metabolism from growth and development toward defense mechanisms is an important component of drought tolerance of Arabidopsis and of the physiological response to β-CCA. The present study has identified SMR5 and β-CCA as regulators of this phenomenon, hence providing new actors in the process by which plants can balance growth and stress response. Future strategies for resetting the balance between stress resistance and growth to engineer stress-resistant and high-yielding crops require the understanding of how stress signaling regulates plant growth (82). Induction of SMRs appears to be a piece of the puzzle, which links the plant cell division machinery with the defense mechanisms against stress.

## Materials and methods

### Plant material and growth conditions

WT *Arabidopsis thaliana* (ecotype Col-0), a triple SMR knockout mutant (*smr4 smr5 smr7*), and transgenic lines overexpressing *SMR5* or *SMR4* (*OE:SMR5* and *OE:SMR4*, respectively), provided by L. De Veylder (VIB, Belgium), were used in this study. A number of additional *OE:SMR5* and *OE:SMR4* transgenic lines have also been produced in the frame of this study (see below). Plants were grown on potting soil for 5 or 6 weeks in short-day conditions (8/16 h, light [150 µmol photons m^−2^ s^−1^]/dark) under controlled environmental conditions in phytotrons of the Phytotec platform (BIAM, CEA/Cadarache), as previously described (13, 16). The size of the pots (one plant per pot) was 5.5 × 5.5 × 5 cm (about 150 cm^3^). In most experiments, β-CCA was applied to the plants through the roots by watering the pots with a 1.5 mM solution of β-CCA (25 mL per plant). The β-CCA solution (1.5 mM, pH 5) was produced as previously described (13) and as described in Supplementary material.

Arabidopsis was also grown in vitro on Agar in Petri dishes, as described in Supplementary material.

### β-CCA analyses

β-CCA was measured in plant tissues by gas chromatography coupled with mass spectrometry (GC-MS) as described in detail in Supplementary material.

### Water stress treatments

Water withdrawal was applied to 5-week-old plants by stopping watering for 8 to 15 days. The RWC of the leaves and the soil moisture were measured as described in Supplementary material.

Osmotic stress was applied to seedlings grown in vitro as described in (83). Polyethylene glycol (PEG 8000)–infused medium was prepared by overlaying 60 mL of PEG solution (200 g dissolved in 1 L MS/10) on 40 mL of solidified growth medium for at least 12 h. Control plates were overlayed with MS/10. The excess liquid solution was then poured off, and 3-day-old Arabidopsis seedlings were transferred on the Agar medium using sterile nylon strips.

### Stomatal conductance

Stomatal conductance of fully expanded leaves was measured on 6-week-old plants using a hand-held AP4 porometer (Delta-T Devices). Measurements were carried out on the abaxial leaf side in the middle of the light period following the instruction manual of the porometer. The apparatus was let to equilibrate in the phytotron for 2 h before measurements.

Plant transpiration was also estimated by IR imaging. Low relative humidity (45 ± 5%) and low wind speed were applied the day before IR thermographic imaging to ensure optimal contrast between lines. Images were acquired using a FLIR IR camera of the A600 series. The pixel resolution of the detector was 640 × 480, and the spectral range was 7.5–14 µm.

### Cuticular transpiration

Freshly cut Arabidopsis rosettes were immediately sealed with vacuum grease on the cut root collar. The rosettes were then placed on a tripod on the weighing pan of a precision balance and were let to dehydrate in complete darkness. The rosette weight was automatically measured every 3 min. At the end of the experiment, the plant was placed in an oven at 70°C to determine the dry weight.

### Microscopic analyses

Roots were stained with 0.01% (w/v) polycationic stain ruthenium red (Sigma Aldrich) for 10 min and observed with an Axio Zoom V-16 (Zeiss) microscope.

Confocal laser scanning microscopy imaging was performed with a Zeiss LSM780 or LSM980 confocal microscope. Excitation and detection windows were set at 514 and 650–700 nm, respectively, for propidium iodide and 488 and 500–550 for fluorol yellow. Fluorol yellow staining was performed as described in (84). Roots were incubated in a freshly prepared solution of 0.01% (w/v) fluorol yellow (Santa Cruz) in lactic acid (85%) at 70°C for 30 min. Afterwards, plants were washed three times in water. For propidium iodide staining, roots were incubated in a fresh solution of 10 µg L^−1^ for 5 min and then rinsed in water for 10 min.

The Arbidopsis lines with GUS (β-glucuronidase) marker (25) and the method for GUS staining are described in detail in Supplementary material.

### qRT-PCR

Total RNA extraction and quantitative PCR were performed as described in detail in Supplementary material.

### Plasmid construction and plant transformation

Supplementary lines of *SMR* overexpressors were constructed as described in Supplementary material.

### RNAseq

Root tips (5-mm length) were harvested from Arabidopsis seedlings grown in vitro for 6 days. Total RNA was extracted by using Direct-zol RNA MiniPrep Plus (Zymo Research, R2072). Quantity and quality of RNA were assessed respectively using NanoDrop2000 (Thermo Scientific) and Qubit RNA IQ assay kit (Thermo Scientific). Samples were analyzed by BGI Genomics (Hong Kong), providing a stranded mRNA library, 20 M reads/sample, 100 bp paired-end reads (100PE) on DNBseq. Standard bioinformatics analyses were performed by the Doctor Tom platform of BGI Genomics.

### Analysis of polyester monomers and cuticular waxes

Cuticular waxes of rosette leaves and leaf and root cell wall polyesters were analyzed as previously described (46, 85). A description of the protocols is given in Supplementary material.

## Supplementary Material

pgad353_Supplementary_Data

## Data Availability

The data generated in this study are included in the main text and in Supplementary material.

## Preprint

The first version of this manuscript was posted on a preprint.
