# Supplementary material for: The response of Arabidopsis to the apocarotenoid β-cyclocitric acid reveals a role for SIAMESE-RELATED 5 in root development and drought tolerance
Source: PNAS Nexus. 2023 Oct 26;2(11):pgad353. doi: 10.1093/pnasnexus/pgad353 (PMC10638494; doi:10.1093/pnasnexus/pgad353)
Supplement: pgad353_Supplementary_Data [file pgad353_supplementary_data.pdf]

Supplemental material

The response of Arabidopsis to the apocarotenoid β-cyclocitric acid reveals a role for SMR5 in root development and drought tolerance

J. Braat, M. Jaonina, P. David, M. Leschevin, B. Légeret, S. D'Alessandro, F. Beisson and M. Havaux

Materials and Methods

In vitro culture of Arabidopsis seedlings

For *in vitro* cultures on Agar in petri dishes, Arabidopsis seeds were surface-sterilized for 2 min in a solution containing 70% (v/v) ethanol and 0.05% (v/v) sodium dodecyl sulfate, and washed twice with 95% ethanol. Seeds were then grown on vertical plates on 10 times diluted Murashige and Skoog medium (MS/10) containing 0.5% sucrose and 0.8% Agar (w/v) under long-day conditions (16 h/8 h, day/night) at 22°C. The medium was supplemented with 2.5 mL of 0.20 µm-filtered 1.5 mM solution of β-CCA (2.5 ml in 50 ml of liquid medium, corresponding to a final concentration of 75 µM β-CCA, unless specified otherwise) or with ultrapure water (for the controls). Because the growth medium is buffered (pH 5.8), addition of 75 µM β-CCA had no significant effect of its pH. Roots of plants grown on soil or on sand were carefully washed in water before measuring root length and dry weight.

For hypocotyl length measurements, the sterilized seeds were sown on Petri dishes containing the MS/10 medium supplemented with different concentrations of β-CCA. They were put at 4°C overnight, and then transferred to the dark at 24°C for 7 d.

Leaf relative water content and soil moisture

Leaf relative water content (RWC) was measured by weighing leaf disks (fresh weight, FW). The leaf disks were then fully hydrated for 24 h in water and in the dark (turgid weight, TW). Finally, the disks were dried at 70°C for at least 48 h (dry weight, DW). The RWC (in %) was calculated as [(FW-DW)/(TW-DW)] x 100. Soil moisture was measured by weighing a soil aliquot before and after drying in an oven for 48 h (FW and DW, respectively). Soil moisture (in %) was calculated as [(FW-DW)/FW] x100.

β-cyclocitric acid analyses

1 mL of 5 % H_2SO_4 (v/v) in methanol was added to 50 mg of plant tissue and heated at 90°C for 90 min. The sample was cooled down to room temperature and vortexed. 500 µL of hexane was then added, followed by 1.5 mL 0.9% NaCl (w/v). The mixture was vigorously stirred for 40 s with a vortex mixer and centrifuged for 2 min. The upper phase was analyzed by GC-MS (7890A gas chromatograph and 5975C mass spectrometer; Agilent Technologies) using a polar OPTIMA WAX column (30 m 3 0.25 mm 3 0.5 mm, Macherey-Nagel). The GC conditions are as followed: splitless mode injection; injector temperature, 240°C; oven temperature program: 50°C for 1 min followed by a temperature ramp of 10°C min^{-1} to 300°C, holding this temperature for 2 min. The flow rate of the carrier gas (He) was 1 mL min^{-1}. Mass spectrometer parameters were positive mode and single ion monitoring mode. β-CCA was quantified on ions m/z 123 and m/z 135.

To measure the *in vivo* conversion of β-CC into β-CCA, seedlings were grown on partitioned Petri dishes, as described in Dickinson et al. (2019). 25 µM β-CC was added to the Agar medium in one of the partition, and seedlings were grown in the other partitions (exposure to volatile β-CC). Seedlings were also exposed directly to 750 nM β-CC (direct contact of roots with β-CC).

Microscopic analyses

The following homozygous GUS lines were used: a) a cyclin (CYC) gene fused with the GUS reporter for the translational reporter lines *CYCB1;1-GUS*, *CYCB1;2-GUS*, *CYCB3;1-GUS*, *CYCA2;3-GUS*, *CYCA3;1-GUS, CYCA3;2-GUS;* b) the promoter of *CYC-D/CCS52A1/SIAMESE/SIAMESE-RELATED* genes fused to the GUS marker for the transcriptional reporter lines *pCYCD3;3::GUS, pCYCD6;1::GUS*, *pCCS52A1::GUS*, *pSIM::GUS*, *pSMR4::GUS*, *pSMR5::GUS* and *pSMR7::GUS.* Seeds were grown in Petri dishes containing MS/10 medium. After 6 d, the seedlings were incubated at 37°C with X-glucuronidase solution (1mg/mL) containing 10 mM phosphate (pH 7), 10% (w/v) Triton X-100, 1 M potassium ferricyanide and 100 mM potassium ferrocyanide for 4h for *pSMR4::GUS*, *pSMR5::GUS* and *pSMR7::GUS* or overnight for the other lines. The staining was stopped with 75% (v/v) ethanol and mounted between slides and coverslips. GUS expression was observed with an Axio Zoom V16 (Zeiss) microscope.

qRT-PCR

Total RNA was using Direct-Zol RNA MiniPrep (Zymo Research) and treated with the RNase-free DNase Set (Zymo Research) according to the manufacturer's instructions. Quantity of RNA was assessed using a NanoDrop2000 (Thermo Scientific, USA). Reverse transcription was performed on 400 ng of total RNA using qScript cDNA SuperMix (Quantabio). Quantitative PCR was performed on a 480 Light Cycler thermocycler (Roche) using the manufacturer's instructions, using SYBR Green I Master (Roche) with 10 µM primers and 0.125 µL of RT reaction product in a total volume of 5 µL per reaction. The qPCR program was : 95 °C for 10 min, then 45 cycles of 95 °C for 10 s, 60 °C for 10 s and 72 °C for 10 s. The specific primers for PCR amplification, designed using the NCBI Primer designing tool, are listed in Supplemental Table 1. *UPL7* was used as reference gene for data normalization.

Plasmid construction and plant transformation

Transgenic lines *OE:SMR5* and *OE:SMR4* were generated by overexpressing the *SMR* genes under the control of the 35S promoter. We received *E. coli* strains containing SMR open reading frames cloned into the pDONR221 entry vector by BP recombination cloning from Dr. Lieven De Veylder (VIB Belgium). The *SMR* open reading frames were then transferred into the pB2GW7 destination vector by recombinational cloning with Plant Gateway vectors (Karimi et al. 2007). All constructs were transferred into the *Agrobacterium tumefaciens* C58C1RifR strain. Transgenic plants in the T_2 generation with T-DNA insertion at a single locus were selected by phosphinotricin resistance (10 mg L^{-1}), and T_3 homozygotes were used for all analyses. *SMR* gene expression was quantified in the overexpressing lines by qRT-PCR (see above) using *UCP* (supplemental Table 1) as internal control for data normalization.

Analysis of polyester monomers and cuticular waxes

Rosette leaves (250 mg fresh weight) of 4 week-old plants grown on soil were dipped in chloroform for 30 s and the cuticular waxes analyzed as previously described (Jakobson et al. 2016). Leaves were previously photographed to measure total area. For root polyester analysis, 100 mg (fresh weight) of roots were harvested from seedlings grown for 8 d on solid Agar medium and added to boiling isopropanol (85°C) containing 0.01% (w/v) butylated hydroxytoluene and heated for 10 min. For leaf polyester analysis, the same isopropanol procedure was applied to the leaves used for the wax analysis immediately after the chloroform remaining on the leaves had evaporated. Leaf and root cell wall polyesters were delipidated and depolymerized as previously described in detail (Li-Beisson et al. 2013). Briefly, the plant material quenched in isopropanol was delipidated and the cell wall polyesters were broken down to fatty acid methyl esters (FAMEs) using sulfuric acid-catalyzed depolymerization, the FAMEs were derivatized by acetylation and separated and quantified by gas chromatography coupled to mass spectrometry (GC-MS). GC-MS analysis was performed using conditions previously described (Jakobson et al. 2016).

References:

Dickinson AJ, Lehnera K, Mid J, Jiad K-P, Mijara M, Dinneny J , Al-Babili S, Benfey, PN. 2019. β-Cyclocitral is a conserved root growth regulator. *Proc. Natl. Acad. Sci. USA* 116: 10563-10567.

Jackobson L, Lindgren LO, Verdier G, Laanemets K, Brosché M, Beisson F, Kollist H. 2016. BODYGUARD is required for the biosynthesis of cutin in Arabidopsis. *New Phytol.* 211: 614-626.

Karimi M, Depicker A, Hilson P. 2007. Recombinational cloning with plant getaway vectors. *Plant Physiol.* 145: 1144-1154.

Li-Beisson Y et al. 2013. Acyl-lipid metabolism. *The Arabidopsis Book* 11:e0161.

Supplemental Figures

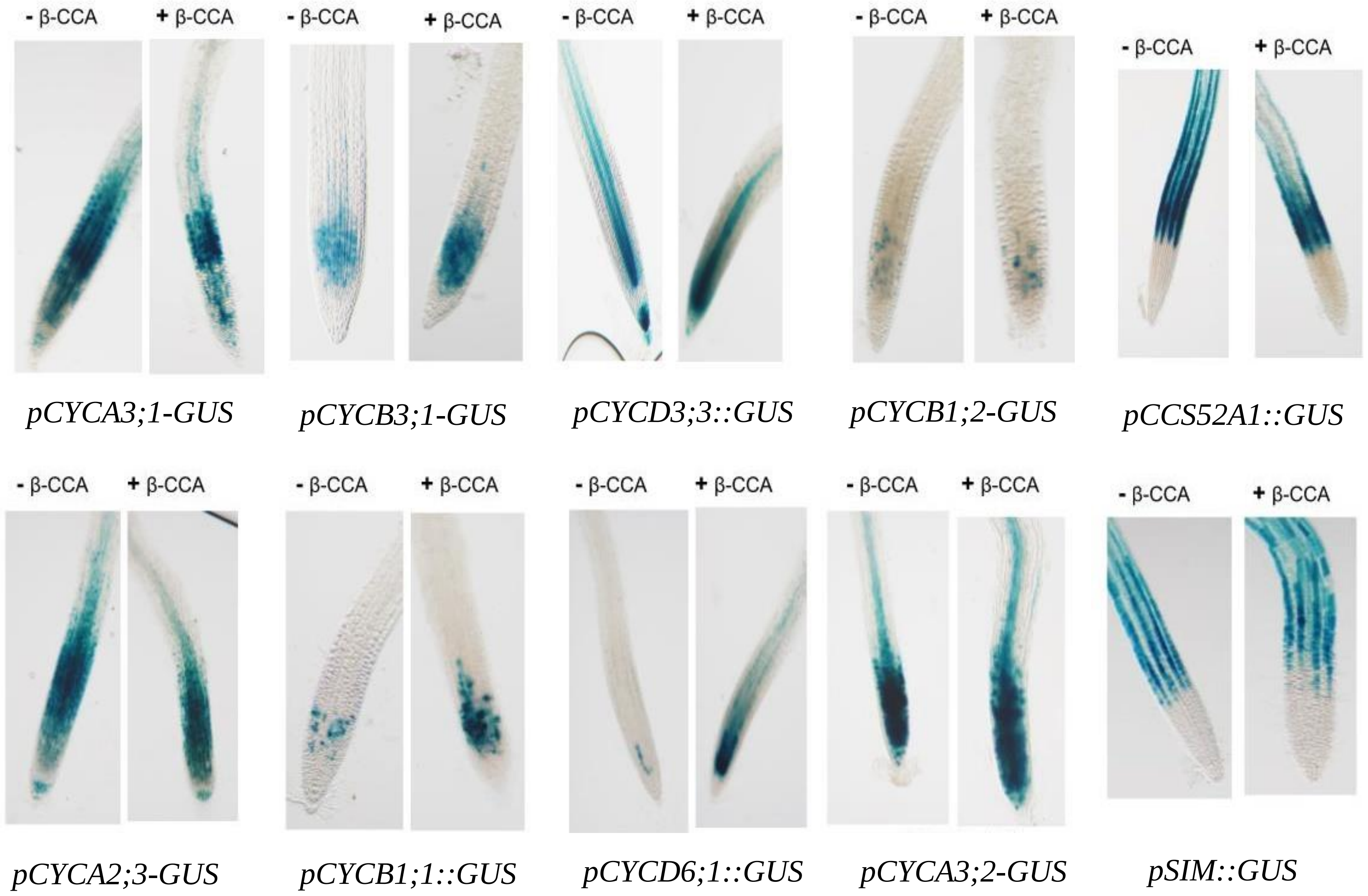

Fig. S1. GUS coloration of root tips of the translational GUS reporter lines *CYCB1;1-GUS*, *CYCB1;2-GUS*, *CYCB3;1-GUS*, *CYCA2;3-GUS*, *CYCA3;1-GUS, CYCA3;2-GUS* and the transcriptional reporter lines *pCYCD3;3::GUS, pCYCD6;1::GUS*, *pCCS52A1::GUS*, *pSIM::GUS*. Seedlings were exposed to 0 or 75 µM β-CCA in the growth medium.

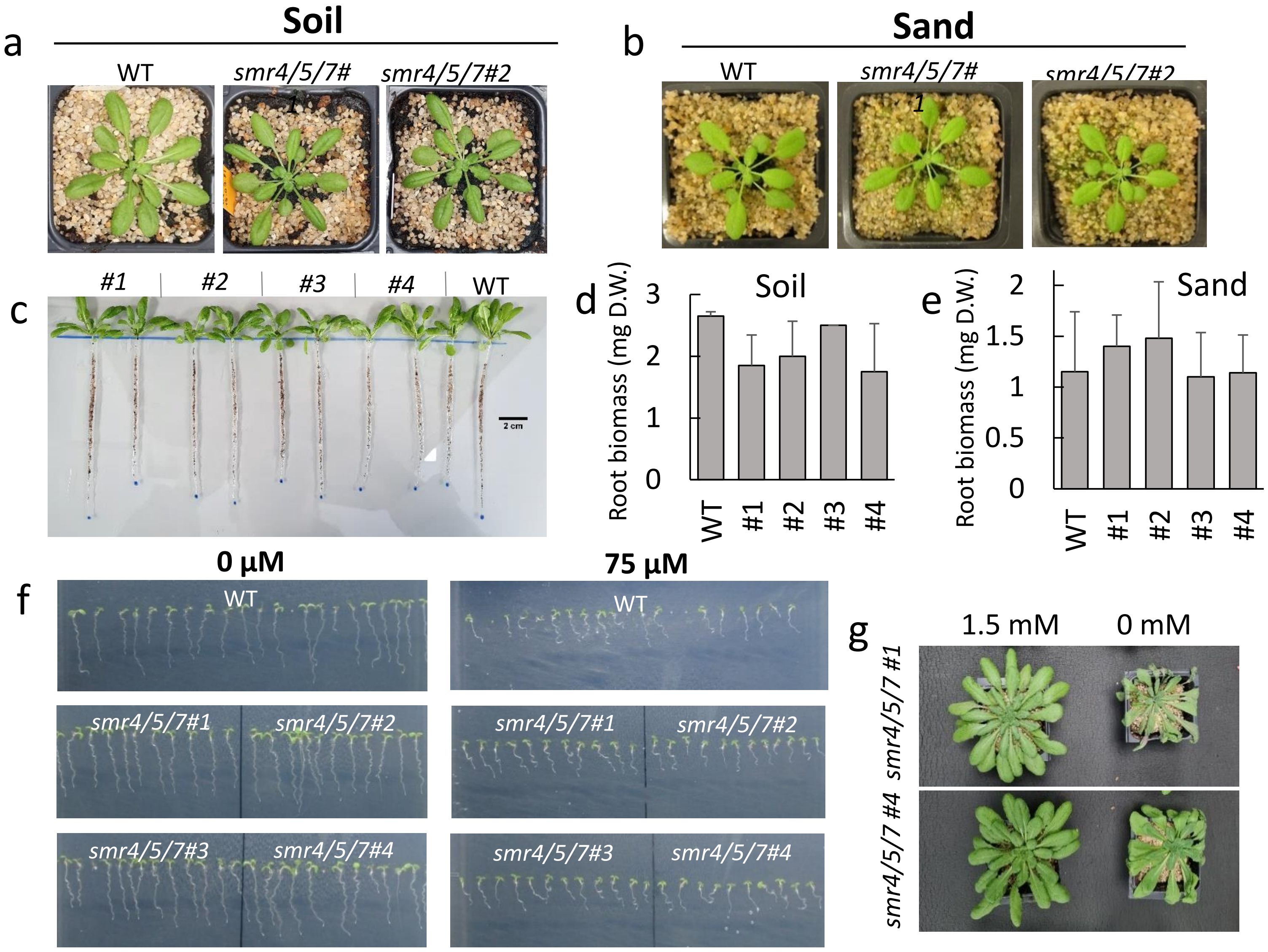

Fig. S2. Root growth is not affected in the SMR triple mutant *smr4 smr5 smr7*. Representative rosettes of *smr4 smr5 smr7* triple mutants grown A) on soil for 5 weeks or B) on sand for 4 weeks. C) Root system of plants grown on soil for 5 weeks. D) and E) Roots were thoroughly washed under running water and then dried to measure dry weight (D.W.). Values are means + SD (n=2) in D) (n=5) in E). f), WT and mutants were grown *in vitro* with 0 or 75 µM β-CCA to check root length. G) Drought response of soil-grown triple *smr* mutants pretreated with 0 or 1.5 mM β-CCA.

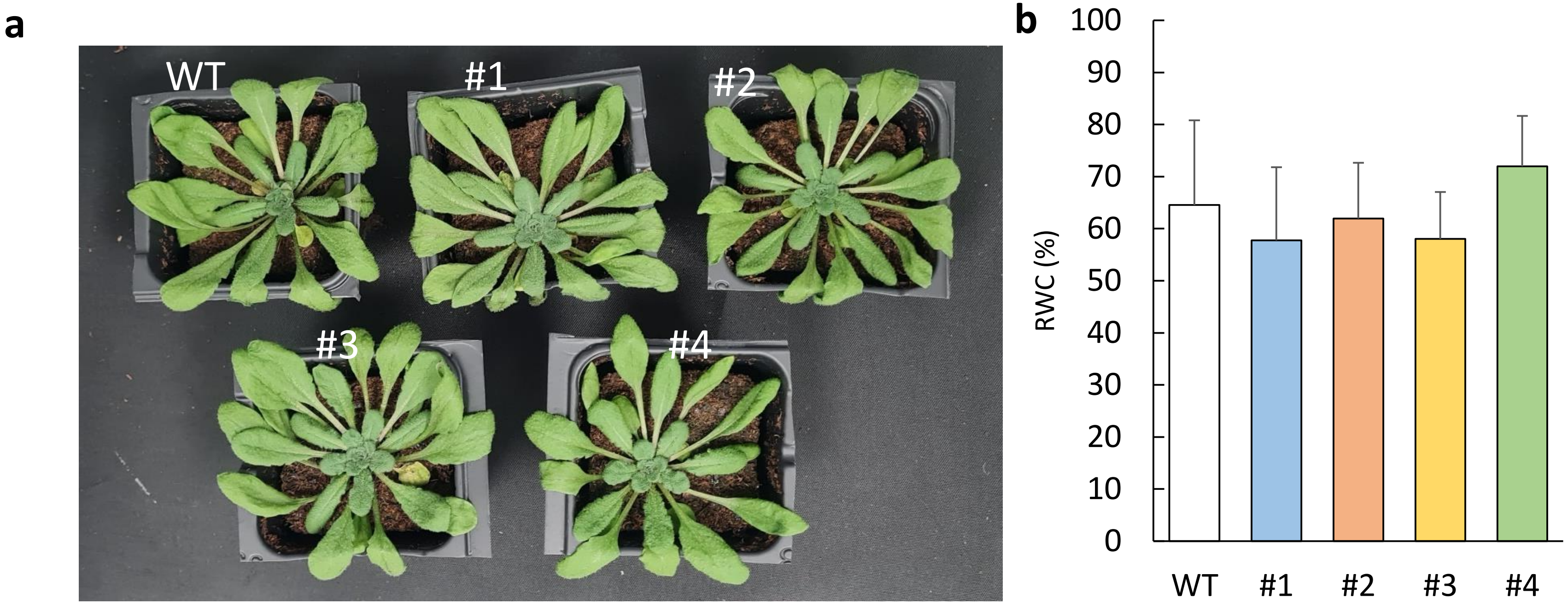

Fig. S3. Relative drought tolerance of WT and the triple mutant *smr4 smr5 smr7*. Plants were submitted to water deprivation for 8 d. A) Picture of stressed plants (WT and 4 triple mutant lines). B) Leaf RWC. Data are mean values of 2 separate experiments with 4-5 plants per condition in each experiment, + SD.

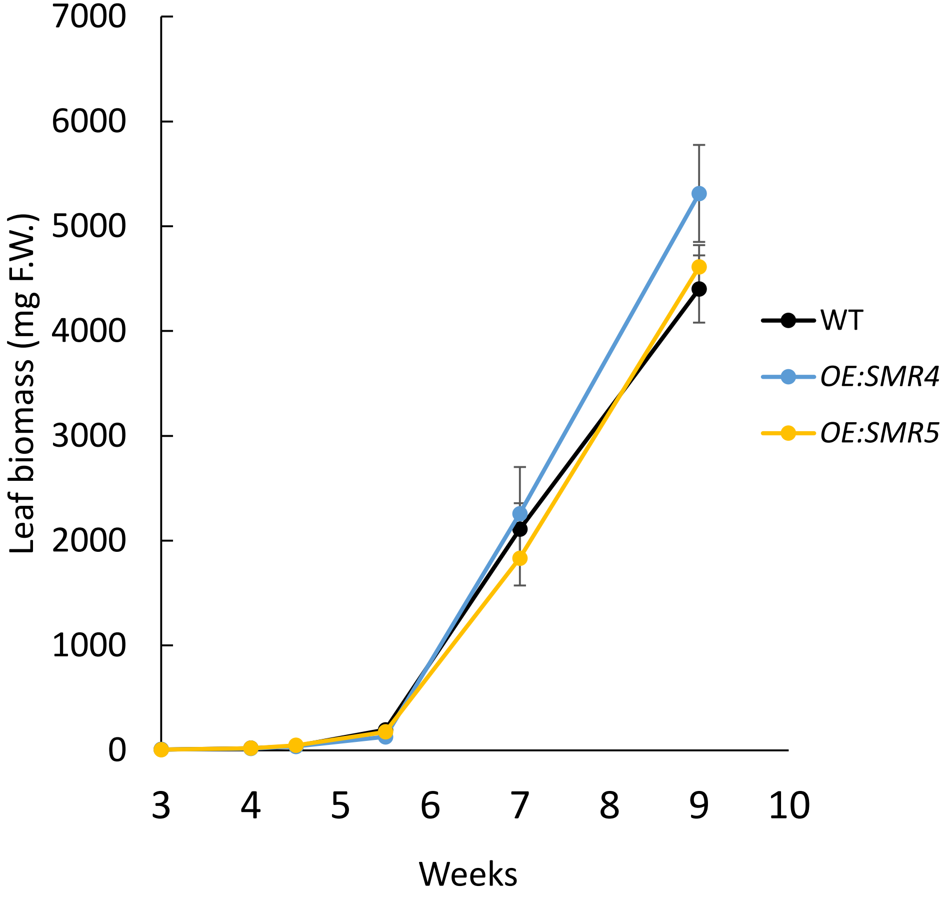

Fig. S4. Growth curves of soil-grown WT, *OE:SMR5* and *OE:SMR4* Arabidopsis plants.

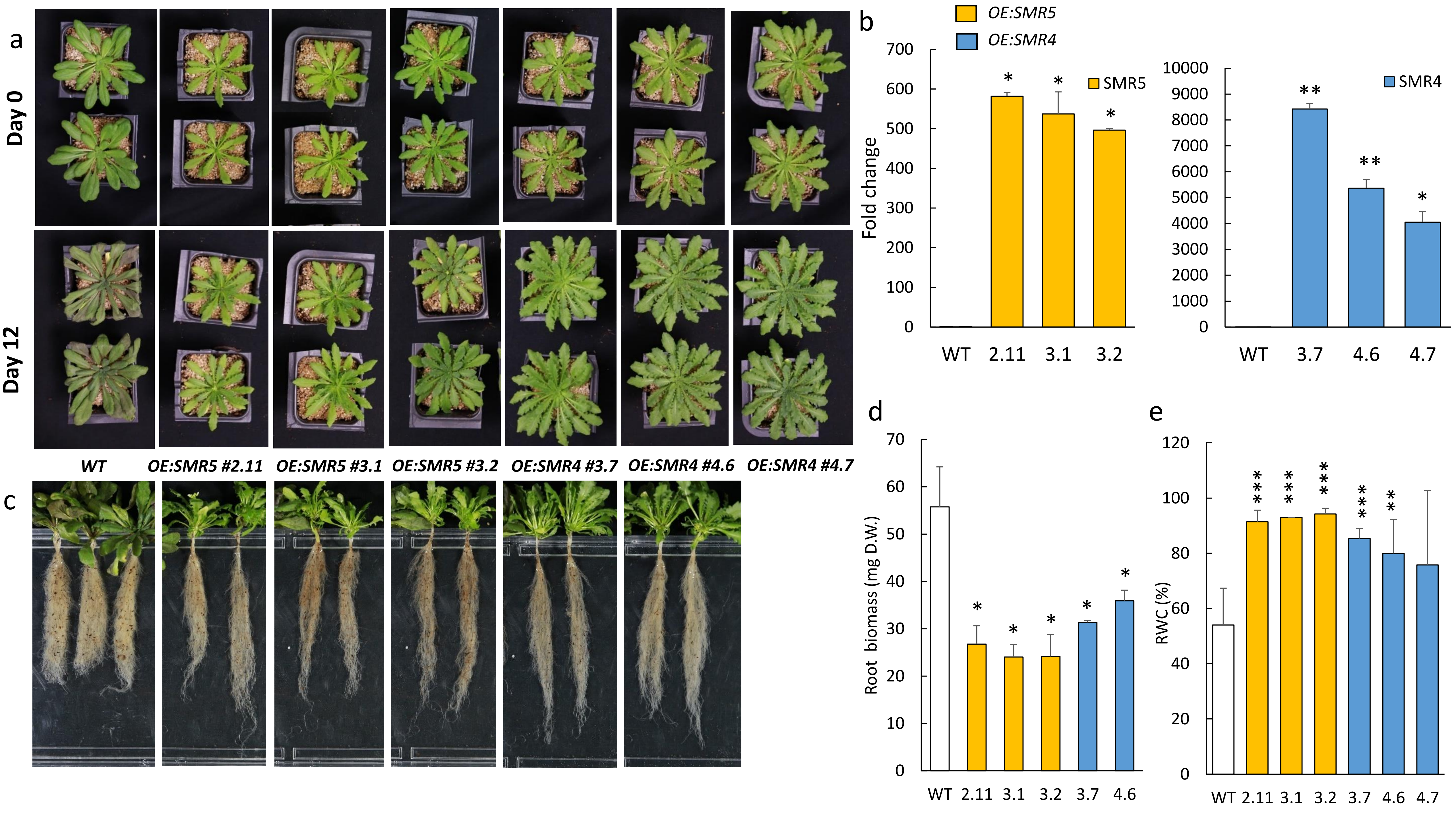

a
Day 0
Day 12
WT
OE:SMR5 #2.11
OE:SMR5 #3.1
OE:SMR5 #3.2
OE:SMR4 #3.7
OE:SMR4 #4.6
OE:SMR4 #4.7
b
OE:SMR5
OE:SMR4
Fold change
SMR5
SMR4
WT 2.11 3.1 3.2
WT 3.7 4.6 4.7
c
d
Root biomass (mg D.W.)
WT 2.11 3.1 3.2 3.7 4.6
e
RWC (%)
WT 2.11 3.1 3.2 3.7 4.6 4.7

Fig. S5. Drought response of a series of independent *SMR4*- and *SMR5*-overexpressing transgenic lines**.** A) Picture of *OE:SMR4* and *OE:SMR5* lines before and after 12 d of water deprivation. B) Expression levels of *SMR4* and *SMR5* genes in the respective *OE:SMR* lines. Expression in WT was set to 1. C) and D) Pictures and dry weight of the root system of WT and *OE:SMR* lines after 12 d of drought stress. Data are mean values of 3 experiments + SD. E) Leaf RWC of plants after 12 d of water deprivation. Data are mean values of at least 3 experiments + SD. *, **, ***, different from WT at $P < 0.05$, 0.01 and 0.001, respectively (Student's t-test).

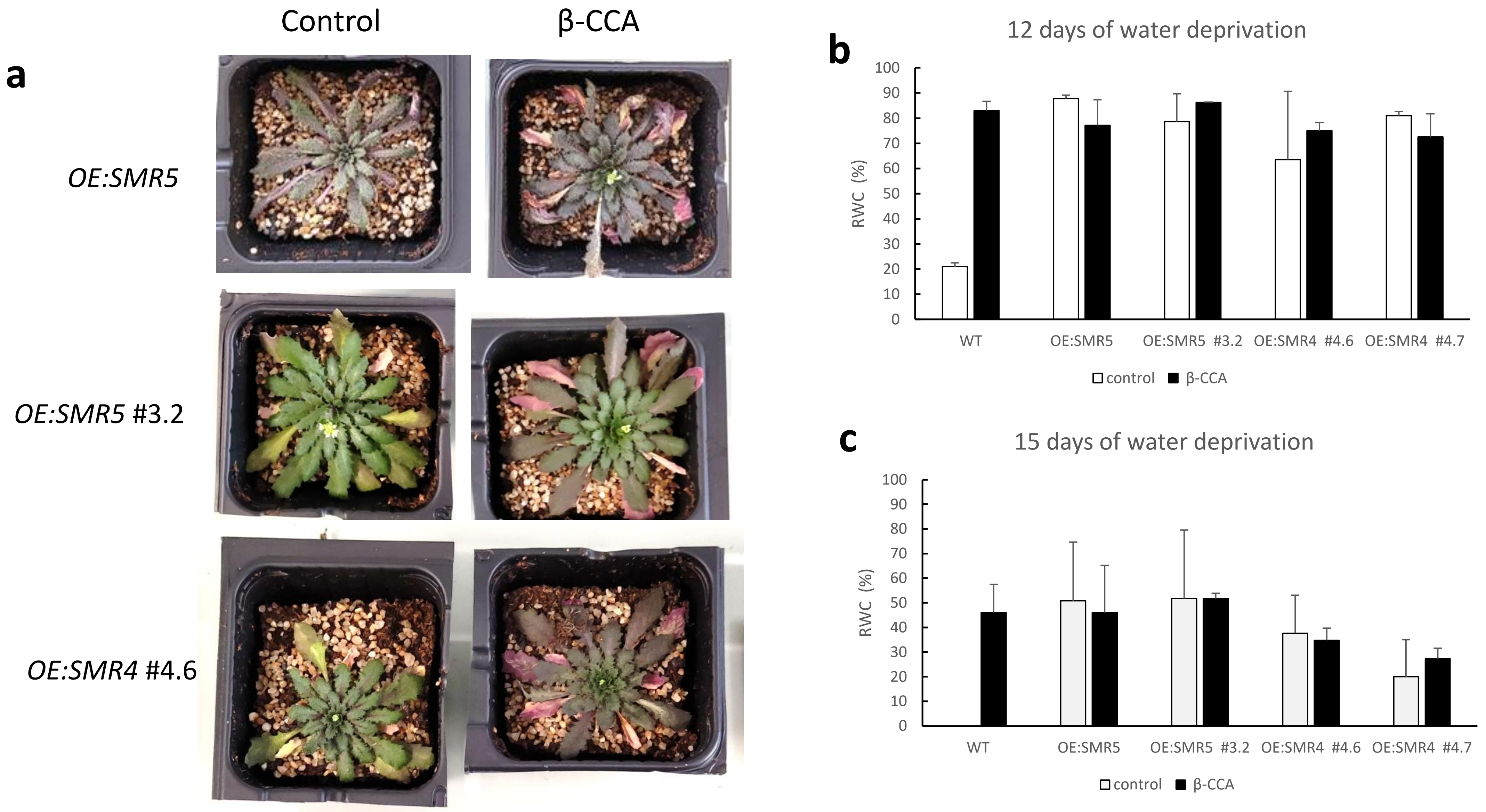

Fig. S6. A) Typical examples of *OE:SMR* plants (pretreated with 0 or 1.5 mM β-CCA) after 15 d of water deprivation. b) RWC of WT and *OE:SMR* plants after (B) 12 d or (C) 15 d of water deprivation. Data are mean values of 3 separate experiments + SD.

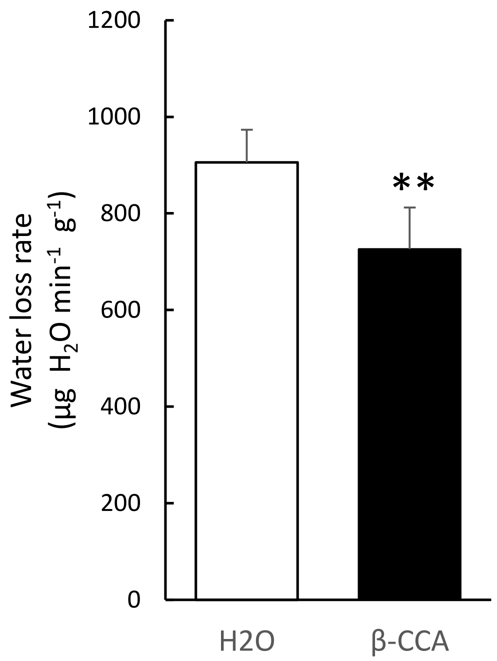

Fig. S7. Effect of 1.5 mM β-CCA on leaf cuticular transpiration. Plants were pre-treated with 0 or 1.5 mM β-CCA for 4 d, then rosettes were excised, placed in the dark, weighed at regular intervals of time to measure water losses. Cuticular transpiration was calculated as in Fig. 4f,g. **, different from H_2O at $P < 0.01$ (Student's t test), n=4.

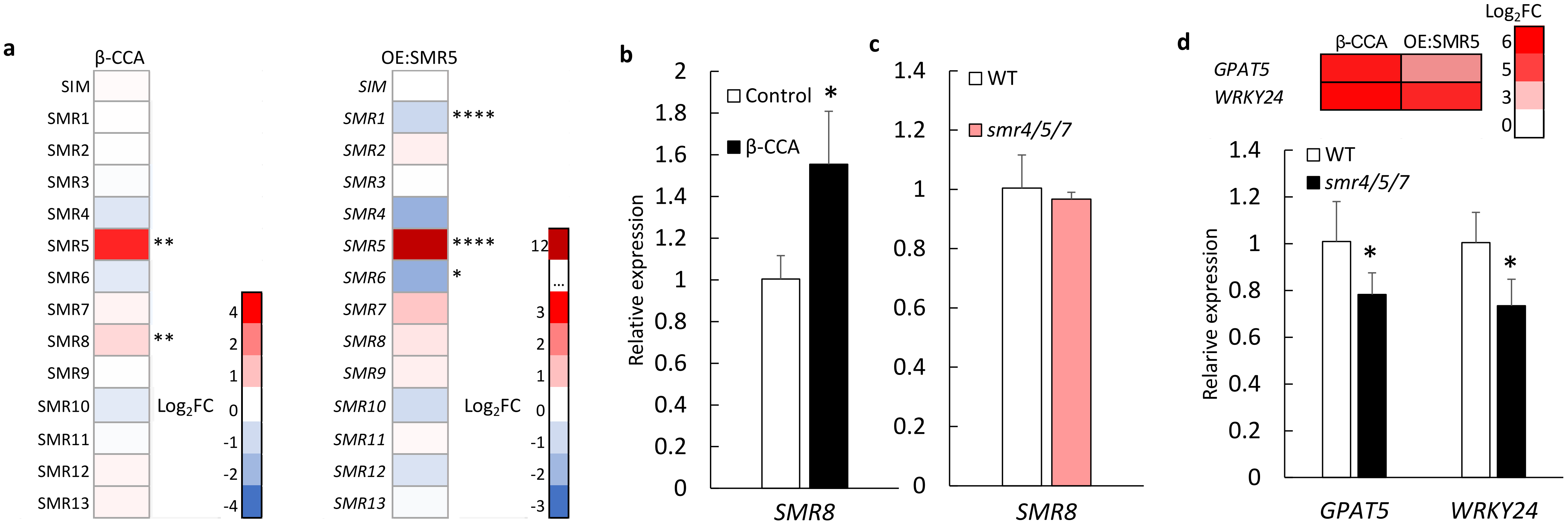

Fig. S8. A) Effect of 1.5 mM β-CCA or *SMR5* overexpression on the expression of *SIM/SMR* genes analyzed by RNAseq. Significant gene expressions are marked with stars with *, **, ***, **** indicating Q values (P adjusted) < 0.05, 0.01, 0.001, 10^{-10}, respectively. B) Relative expression of *SMR8* in roots of β-CCA-treated WT seedlings. C) Relative expression of *SMR8* in roots of *smr4 smr5 smr7* triple mutant seedlings in control conditions. D) Relative expression of *GPAT5* and *WRKY24* genes in *smr4 smr5 smr7* triple mutant seedlings in control conditions. Both genes are strongly inducible by β-CCA and *SMR5* overexpression (top)

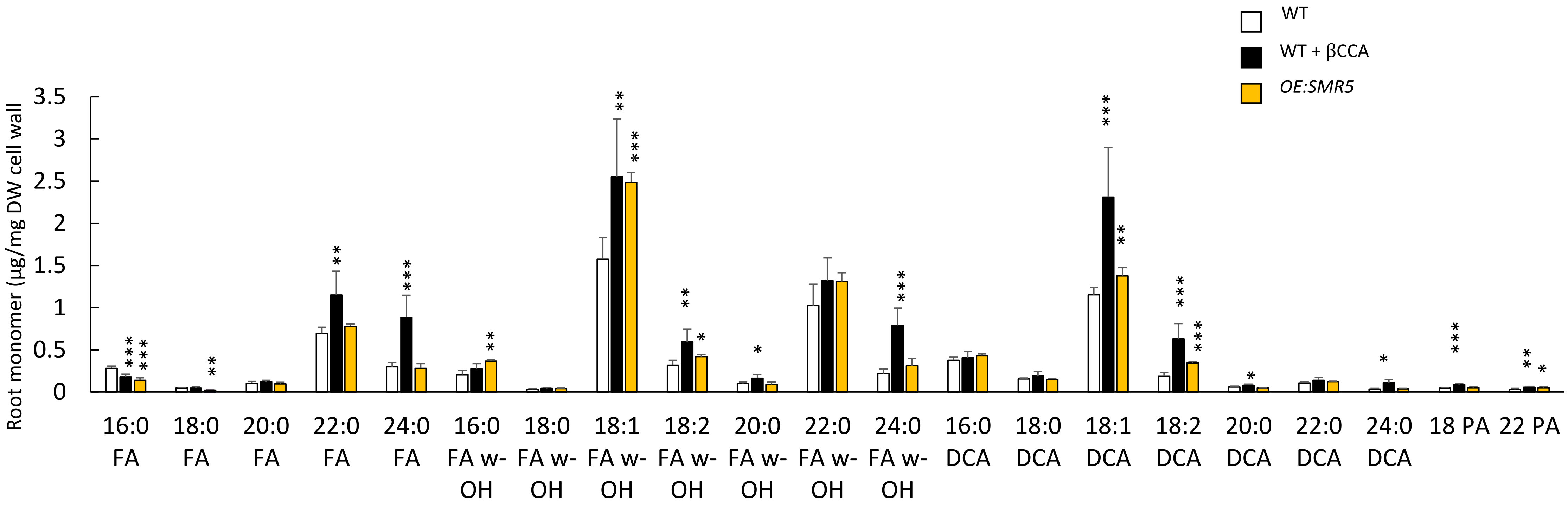

Fig. S9. Profile of root suberin monomers in WT, 75 µM β-CCA-treated WT and *OE:SMR5* seedlings. Plants were grown in vitro of Agar solid medium. The values are means of 3 to 6 biological replicates. *, **, ***, different from WT at $P < 0.05$, 0.01 and 0.001, respectively (Student's t-test). FA, fatty acids; DCA, α,ω-dicarboxylic acids,; FA ω-OH, ω-hydroxy fatty acids; PA, primary alcohols.

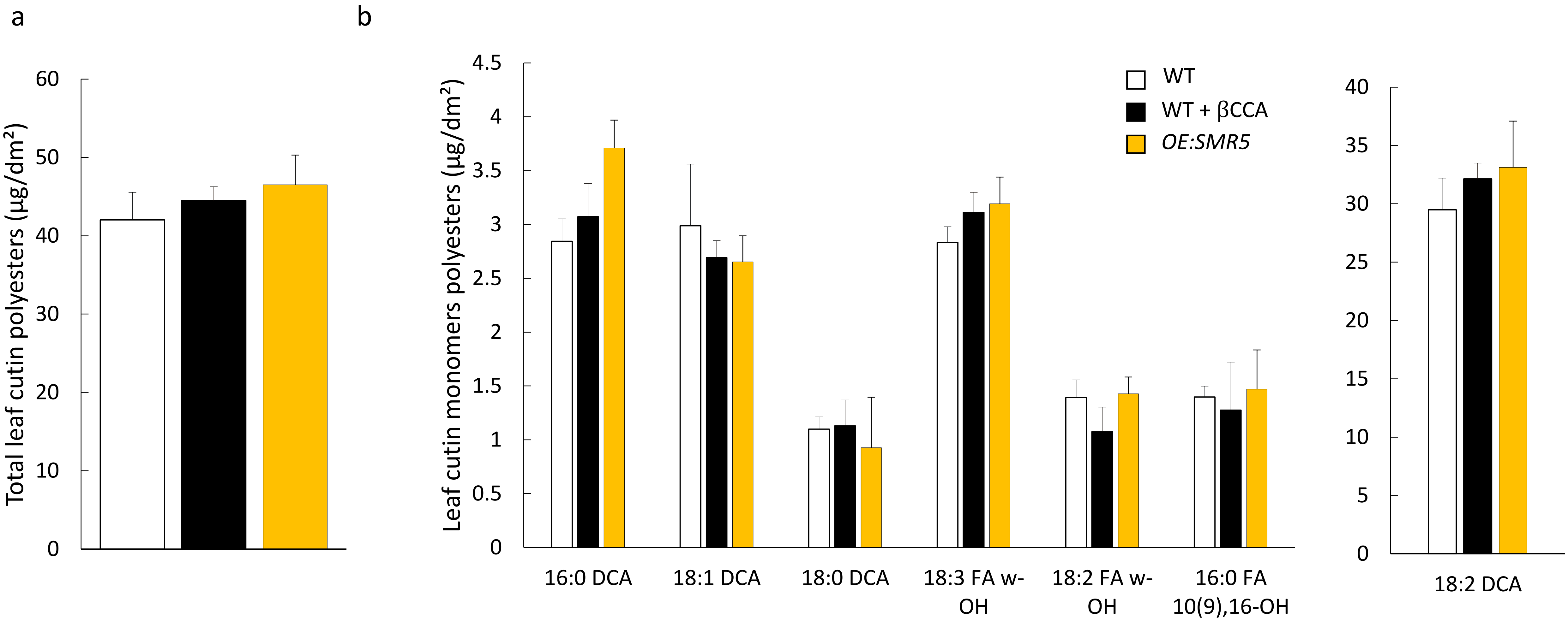

Fig. S10. Leaf cuticle in Arabidopsis plants treated with 1.5 mM β-CCA or overexpressing *SMR5*. A) Total leaf cutin polyesters is calculated as the sum of each monomer shown in B). DCA, α,ω-dicarboxylic acids,; FA ω-OH, ω-hydroxy fatty acids.

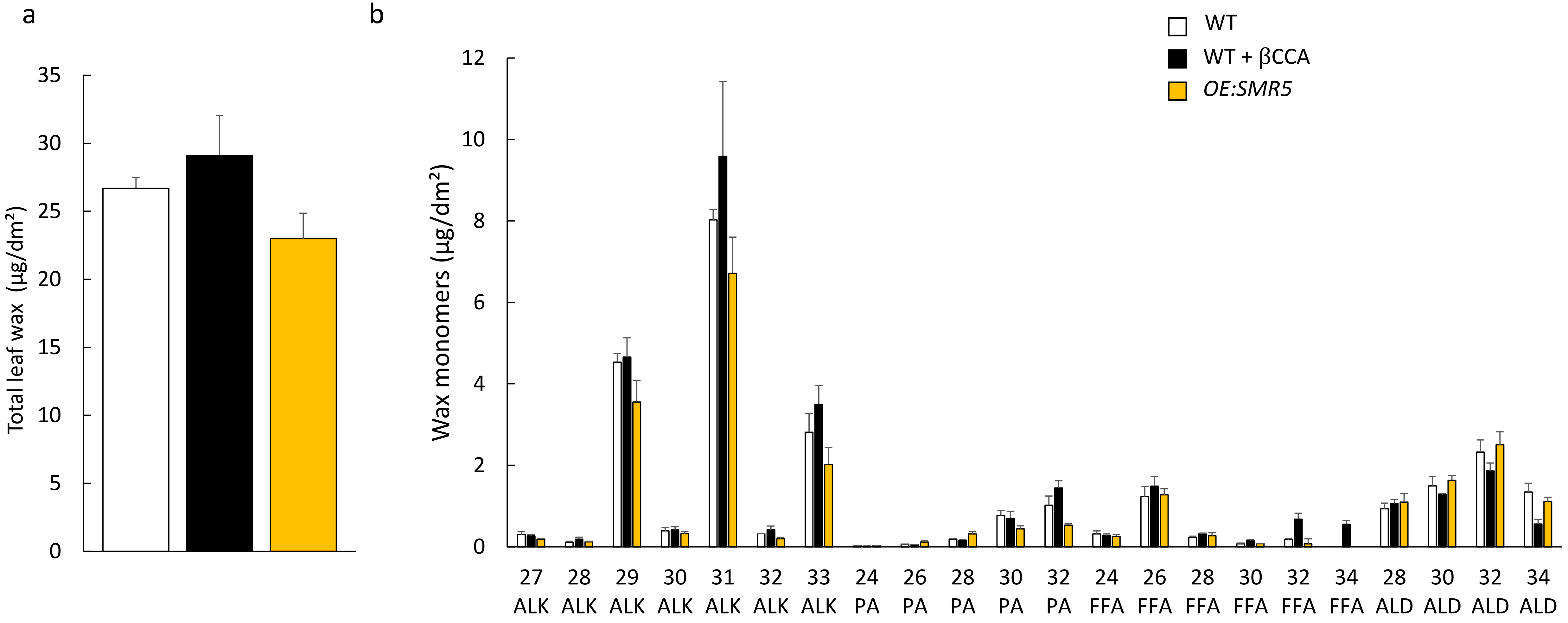

Fig. S11. Leaf waxes in Arabidopsis plants treated with 1.5 mM β-CCA or overexpressing *SMR5*. A) Total leaf wax amount is calculated as the sum of each monomer shown in B). ALK, n-alkanes; PA, primary alcohols; FFA, free fatty aids; ALD, aldehydes.

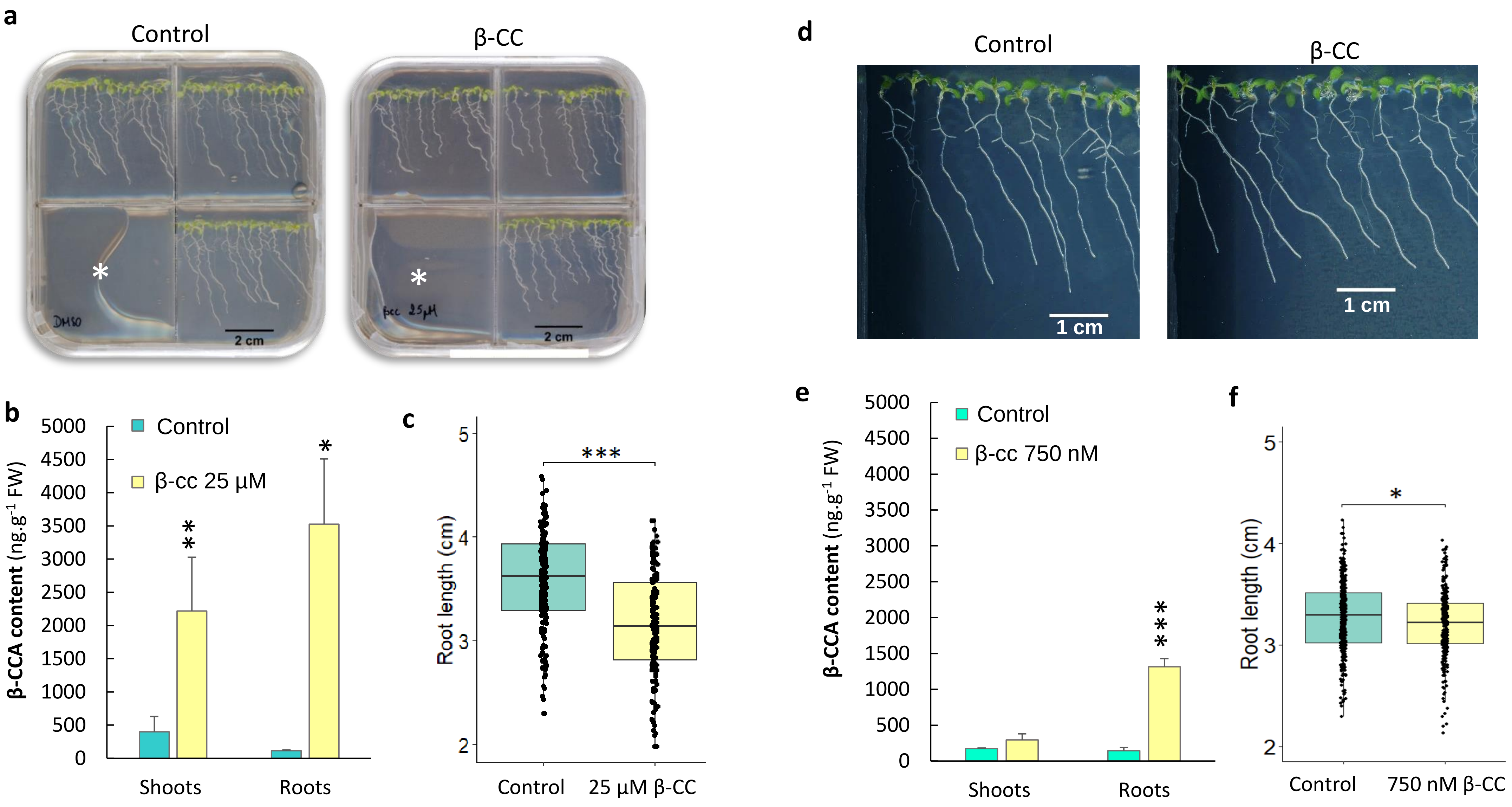
a
Control
β-CC
DMSO
βcc 25μM
2 cm
2 cm
d
Control
β-CC
1 cm
1 cm
b
β-CCA content (ng.g-1 FW)
5000
4500
4000
3500
3000
2500
2000
1500
1000
500
0
Control
β-cc 25 μM
**
*
Shoots
Roots
c
Root length (cm)
5
4
3
2
***
Control
25 μM β-CC
e
β-CCA content (ng.g-1 FW)
5000
4500
4000
3500
3000
2500
2000
1500
1000
500
0
Control
β-cc 750 nM
***
Shoots
Roots
f
Root length (cm)
5
4
3
2
*
Control
750 nM β-CC

Fig. S12. A) Representative pictures of seedlings exposed to 25 µM volatile β-CC in partitioned Petri dishes, as in Dickinson et al. (2019, Ref. 20). The partition marked with a white star contained either 25 µM β-CC or 25 µM DMSO (control) in MS/10 medium. Seeds were plated on the three other partitions on MS/10 growth medium. There was no contact between the partitions, so seedlings were exposed to volatile β-CC only. Plates were tightly sealed with parafilm. B) β-CCA content in roots and shoots of seedlings pictured in A). Values are means of 5 biological replicates + SD. C) Root length of seedlings pictured in A), n=150. D) Representative pictures of seedlings exposed to 750 nM β-CC diluted in MS/10 media. As described in Dickinson et al. (2019), seeds were plated directly on the medium containing 750 nM β-CC or 750 nM DMSO. Plates were sealed tightly with parafilm. E) β-CCA content in root and shoot of seedlings pictured in D). Values are means of at least 3 biological replicates + SD. F) Root length of seedlings pictured in D), n≈300. *,**,*** different from Control at $P < 0.05, 0.01, 0.001$, respectively (Student's t-test).

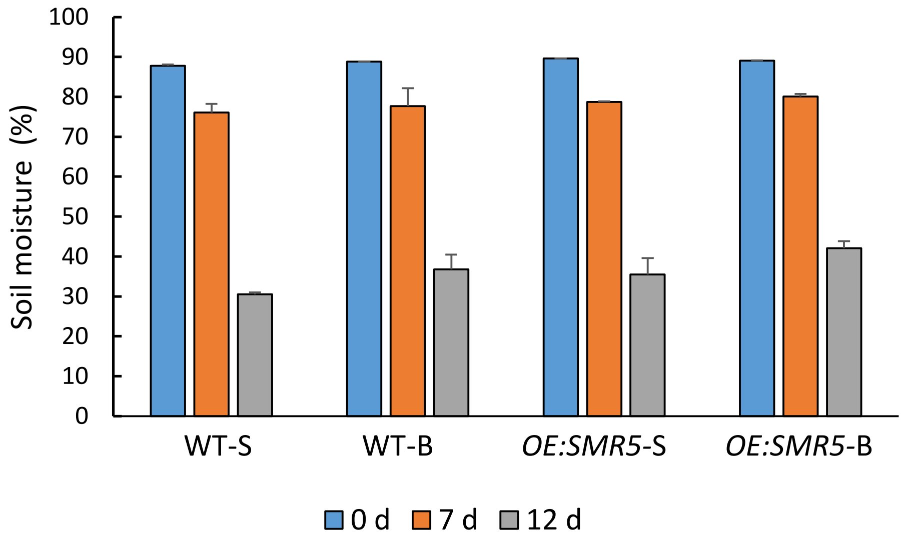

Fig. S13. Soil moisture at the top (WT-S and *OE:SMR5*-S) and the bottom of the pots (WT-B and *OE:SMR5*-B) during water stress. WT and *OE:SMR5* plants were submitted to water deprivation for 0, 7 and 12 d.

Supplemental Table 1

List of primers used for qRT-PCR

**AT5G02220**	*SMR4*	For: GAGGAAGACGGAGATGGCGG<br>Rev: AAAGTACCCGTTCCTCGGCG
**AT1G07500**	*SMR5*	For: ACGACGGAGATACGGTGACG<br>Rev: CTCACCGGAGGTGGACAAGG
**AT3G27630**	*SMR7*	For: AGCCGGTGAAGACGAAACTC<br>Rev: CGCCGTGGGAGTGATACAAA
**AT3G53090**	*UPL7*	For: CTTCTGGGAGGTCATGAAAGG<br>Rev: CTCCAATAGCAGCCCAAAGAG
**AT4G26410**	*UCP*	For: CAGTTCCGCTCTATACAGGATTTC<br>Rev: GCGACACCAATCCCAATAGC
**AT1G10690**	*SMR8*	For : CGCCAAAGAAGCGTAAACCG<br>Rev : AAACGGTTTCTAGGTCCGGC
**AT3G11430**	*GPAT5*	For : ATGGCCCGTAATCACACTCC<br>Rev : TCACGTAGACCAACAGTGGC
**AT5G41570**	*WRKY24*	For : AGGTGAGAAAGGGAAGGAGC<br>Rev: CACGTTGCATGTGTGGTACG
